# A comparison of the safety of oral labetalol versus nifedipine to manage hypertension in pregnancy in Australia: a target trial emulation

**DOI:** 10.1016/j.eclinm.2026.104002

**Published:** 2026-06-05

**Authors:** Jessica A. Atkinson, Anthea C. Lindquist, Stephen Tong, Richard J. Hiscock, Anna Forsythe, Hannah G. Gordon, Susan P. Walker, Su Jen Chua, Catherine Cluver, Jenny Myers, Roxanne M. Hastie

**Affiliations:** a Perinatal Epidemiology Group, Department of Obstetrics, Gynaecology, and Newborn Health, University of Melbourne, Melbourne, Victoria, Australia; b Mercy Perinatal, Mercy Hospital for Women, Heidelberg, Victoria, Australia; c SAMRC Extramural Preeclampsia Research Unit, Stellenbosch University, Cape Town, South Africa; d Maternal and Fetal Health Research Centre, Division of Developmental Biology and Medicine, University of Manchester, Manchester, United Kingdom

**Keywords:** Pre-eclampsia, Labetalol, Nifedipine, Hypertensive disorders of pregnancy, Target trial emulation

## Abstract

**Background:**

Hypertensive disorders of pregnancy are among the leading causes of maternal and neonatal morbidity and mortality worldwide. Labetalol and nifedipine are two of the most common oral antihypertensives used to manage these conditions; however, it is not clear whether one medication may offer greater benefit to mothers and babies than the other. The aim of this study was to compare the risks of adverse maternal and neonatal outcomes for oral labetalol versus nifedipine among women with hypertension in pregnancy.

**Methods:**

This was a target trial emulation using linked data, including pregnancy episodes between January 1, 2009 and December 31, 2020 in Victoria, Australia. We included pregnant women with a hypertensive disorder (chronic hypertension, gestational hypertension, or preeclampsia) and prescribed oral labetalol or nifedipine between 11+0- and 36+6- weeks’ gestation. Our co-primary outcomes were: 1) a composite of maternal mortality or serious morbidity; and 2) a composite of neonatal mortality or serious morbidity. All outcomes were assessed from first prescription until 28 days postpartum. Analyses used a doubly robust inverse probability-weighted regression adjustment (IPWRA) model and are reported as adjusted risk ratios (aRR) and risk differences (aRD) with 95% confidence intervals (95% CI). Analyses were based on intention-to-treat approach.

**Findings:**

7416 pregnancies were eligible for inclusion. Of these, 6745 (91.0%) pregnancies received labetalol as a first-line treatment and 671 (9.0%) received nifedipine. After adjusting for confounding factors, nifedipine was associated with a 33% increased risk of the composite maternal outcome (6.6% versus 9.4%; aRR 1.33, 95% CI 1.07, 1.64), and no difference in the risk of the composite neonatal outcome (43.0% versus 46.1%; aRR 0.97, 95% CI 0.89, 1.06). This was largely driven by increased rates of eclampsia (1.6% versus 3.0%), haemolysis, elevated liver enzymes, and low platelet count (HELLP) syndrome (3.0% versus 4.3%), and renal failure (1.0% versus 1.5%) in the nifedipine group. Among secondary outcomes, nifedipine use was associated with an increased risk of iatrogenic preterm birth and need for additional antihypertensives.

**Interpretation:**

Among women with hypertension in pregnancy, nifedipine was associated with an increased risk of poor maternal outcomes and iatrogenic preterm birth when compared with labetalol. Labetalol may have a better safety profile as a first-line therapy for pregnant women with hypertension. Future clinical trials are required to validate these findings.

**Funding:**

This work was supported by a Trevor B Kilvington Bequest, awarded by the University of Melbourne.

**Research in context**

**Evidence before this study**

Hypertensive disorders of pregnancy (including chronic hypertension, gestational hypertension, and preeclampsia) are among the leading causes of maternal and neonatal mortality worldwide. Oral antihypertensives (including labetalol and nifedipine) are often prescribed to lower maternal blood pressure and reduce the risk of complications. A search of the PubMed database from inception until 12 December 2025 for keywords including “hypertension”, “pregnancy”, “preeclampsia”, “labetalol”, and “nifedipine” yielded 86 results. From these, we identified no adequately powered studies to determine whether one oral antihypertensive may confer greater maternal or neonatal benefit than the other in terms of the risk of adverse outcomes.

**Added value of this study**

In this target trial emulation of 7416 pregnancies, oral nifedipine use was associated with a 33% increased risk of severe maternal morbidity when compared with oral labetalol, among women with hypertensive disorders of pregnancy. Oral nifedipine was also associated with an increased risk of iatrogenic preterm birth and requirement for additional antihypertensives. There was no difference between medications in terms of adverse neonatal outcomes.

**Implications of all the available evidence**

Oral labetalol may have a better safety profile as a first-line therapy for pregnant women with hypertension than nifedipine. Future clinical trials are required to validate these findings.

## Introduction

Hypertensive disorders of pregnancy are among the leading causes of maternal and neonatal death worldwide.^1^^,^^2^ They can result in maternal organ damage, neurological dysfunction, impaired fetal growth, and preterm birth.^1^

Antihypertensive medications are widely used to manage blood pressure and reduce the risk of maternal complications.^3^^,^^4^ Labetalol (a combined alpha- and beta-blocker) and nifedipine (a dihydropyridine calcium channel blocker) are the most commonly prescribed oral antihypertensive agents in Australia during pregnancy.^5^ In Australia, both oral nifedipine and labetalol are eligible for government subsidies under the Pharmaceutical Benefits Scheme.^6^^,^^7^ Both medications are similarly affordable for patients. Both are considered generally safe for the fetus and have relatively mild side-effects, although beta-blockers have been associated with an increased risk of a small for gestational age infant and nifedipine has been associated with an increased risk of neonatal intensive care unit (NICU) admission in the setting of severe hypertension.^8^^,^^9^ What remains unclear is whether they are equally safe in terms of the risk of severe maternal or neonatal morbidity or mortality.^10^

There have only been a handful of randomised trials directly comparing oral labetalol and nifedipine in the setting of non-emergency hypertension.^10^, ^11^, ^12^, ^13^ A 2017 pilot trial showed both medications are effective among pregnant women with chronic hypertension.^11^ Similarly, a secondary analysis of the CHAP (Chronic Hypertension in Pregnancy) trial showed no difference in the risk of their primary composite outcome (superimposed preeclampsia, iatrogenic preterm birth, placental abruption, and stillbirth or neonatal death) between labetalol and nifedipine.^14^^,^^15^ Conversely, a 2015 randomised trial from India showed a slight increase in the rate of stillbirth among hypertensive women treated with nifedipine, compared with labetalol.^12^

However, these previous trials were not able to assess the risk of hard clinical endpoints, such as serious morbidity and mortality, as their primary outcomes. Examining these rare, but severe, outcomes in randomised trials may not be feasible due to the large sample size required.

Target trial emulation of observational data may offer a solution.^16^^,^^17^ Target trial emulation seeks to mimic the conditions of a randomised trial by: (1) addressing a causal question where the exposure (such as labetalol or nifedipine) and specific clinical decision point (time of recruitment and randomisation) can be readily identified; (2) specifying, *a priori*, the inclusion criteria and primary outcomes of the trial; and (3) correcting for differences in the population that may influence the outcome, independent of the exposure (i.e., balancing differences in baseline characteristics between cohorts, as in a clinical trial).^18^, ^19^, ^20^ All steps are first declared in a prespecified statistical analysis plan before the data is seen and applied to the observational data under a set of identifiable assumptions.^18^, ^19^, ^20^ When applied appropriately, this approach has been shown to produce similar results to those of real-world randomised trials.^21^^,^^22^

Utilising population-level linked data, we performed a target trial emulation to compare maternal and neonatal mortality and serious morbidity following oral nifedipine versus labetalol for the treatment of hypertensive disorders of pregnancy. The design of this emulation was informed by the ongoing Giant PANDA randomised controlled trial, which seeks to examine differences in blood pressure between oral labetalol and nifedipine among women with hypertensive disorders of pregnancy.^13^

## Methods

This study is reported as per the Transparent Reporting of Observational Studies Emulating a Target Trial (TARGET) Statement.^23^

### Study design

This study was conducted within a causal inference framework by performing target trial emulation.

To ensure the assumptions underlying our causal approach were robust, we considered our observational data in direct comparison with the conditions of an ‘ideal’ target trial (Table 1). Our target trial was informed by the design of the Giant PANDA (Pregnancy Antihypertensive Drugs: which Agent is best?) randomised trial, which is being conducted in the United Kingdom.^13^ The primary outcomes for the Giant PANDA trial are changes in blood pressure, not severe morbidity or mortality.^13^ However, we used similar inclusion and exclusion, randomisation, and follow-up criteria to that of Giant PANDA, to ensure our design was reflective of a real-world trial. The details of our analytical framework and causal assumptions were outlined in a prespecified statistical analysis plan, which was agreed upon by all authors prior to commencing analysis (Supplement 1).

**Table 1:** Target trial emulation.

Protocol component	Target trial	Emulation using observational data
Eligibility criteria	All women with a hypertensive disorder of pregnancy (chronic hypertension, gestational hypertension, or preeclampsia) requiring antihypertensive treatment between 11 + 0- and 36 + 6-weeks’ gestation.	All women with a hypertensive disorder of pregnancy (chronic hypertension, gestational hypertension, or preeclampsia) who were prescribed oral labetalol and/or nifedipine between 11 + 0- and 36 + 6-weeks’ gestation.
	*Exclusions:* Contraindication to labetalol or nifedipine; taking both medications prior to randomisation.	*Exclusions*: Taking both medications prior to 11 weeks' gestation; received both medications on the same day (unable to be randomised); did not receive either medication (contraindication or non-indication).
Treatment strategy	Start taking allocated antihypertensive at baseline and continue until no longer indicated.	Same as target trial.
Assignment procedures	Randomised at diagnosis of hypertension requiring medical intervention (first dispensation of study drug).	“Randomised” (via inverse probability-weighting) at point of first dispensation of labetalol or nifedipine between 11 + 0- and 36 + 6-weeks’ gestation.
Follow-up period	From baseline (first dispensation of study drug) until 28 days postpartum.	Same as target trial.
Primary outcome	Maternal or neonatal mortality or severe morbidity.	Same as target trial.
Causal contrasts of interest	Relative risk (RR) and risk difference (RD) of each outcome (point estimate and 95% confidence interval [CI]).	Estimand = average treatment effect (ATE), represented as relative risk (RR) and risk difference (RD) of each outcome (ATE 95% CI).
		Estimator model = doubly robust inverse probability-weighted regression adjustment.
Analysis plan	Intention to treat (ITT): All women randomised to labetalol or nifedipine, regardless of discontinuation or crossover of treatment groups.	Intention to treat (ITT): Same as target trial.
	Per-protocol: All women randomised to labetalol or nifedipine who continued allocated treatment until end of trial.	Per-protocol: Same as target trial.

### Study population

The target trial population included all women with access to obstetric services and a confirmed diagnosis of a hypertensive disorder of pregnancy (gestational hypertension, chronic hypertension, or preeclampsia) requiring pharmacological treatment between 11 + 0- and 36+6- weeks’ gestation, without contraindications to either study drug. Exposure beyond 37 + 0 weeks’ gestation was excluded as Society of Obstetric Medicine Australia and New Zealand (SOMANZ) recommends delivery for this cohort from 37 weeks.^24^

For our emulation, the source population included all pregnancy episodes in Victoria, Australia, between January 1, 2009, and December 31, 2020. All women with a confirmed diagnosis of a hypertensive disorder of pregnancy who were prescribed oral labetalol and/or nifedipine between 11 + 0- and 36 + 6- weeks’ gestation were included. Women taking both study drugs prior to 11 weeks' gestation and women who received their first dispensation of both drugs on the same day were excluded (unable to be randomised). Women taking another antihypertensive (e.g., methyldopa) prior to 11 weeks' gestation were eligible to be included if they ceased their other antihypertensive and commenced either labetalol or nifedipine at or beyond 11 weeks’ gestation.

Perinatal and demographic data were obtained from the validated statewide Victorian Perinatal Data Collection (VPDC), and checked for accuracy using the Victorian Births, Deaths, and Marriages registry.^25^ All pregnancies continuing beyond 20 weeks’ gestation are captured in the VPDC registry.

### Exposure, assignment procedures, and follow-up period

Exposure to oral labetalol or nifedipine was determined using the nationwide Pharmaceutical Benefits Scheme (PBS) data collection. The PBS collection contains information on all outpatient pharmacy dispensations of prescription medicines that qualify for government subsidies, including labetalol and nifedipine.^26^ From this database, we ascertained first date of dispensation of oral labetalol or nifedipine between 11 + 0 and 36 + 6 weeks’ gestation, which was deemed the hypothetical point of randomisation.

Data were analysed according to an intention-to-treat protocol. Women who received both oral labetalol and nifedipine during the study period were classified as crossovers and grouped by first medication dispensed. Women taking both medications prior to 11 weeks’ gestation, and women who received both oral labetalol and nifedipine on the same date were excluded as they could not be feasibly randomised in a trial setting.

**Fig. 1:**
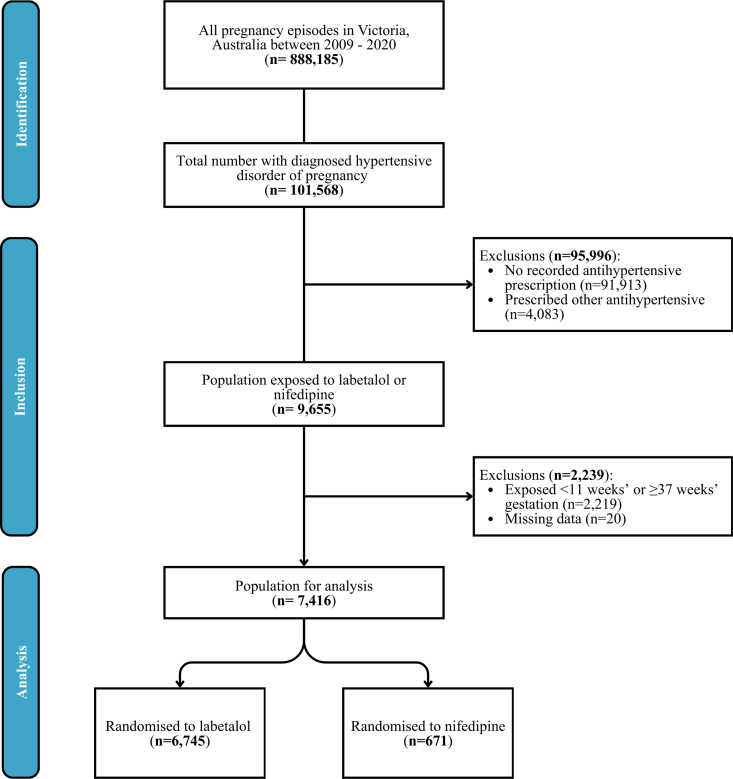
Flowchart of participants.

The follow-up period continued until 28 days postpartum. Pregnancy episodes with missing conception or birth date were excluded from the analysis (Fig. 1). Women who received oral labetalol or nifedipine during the study period without a confirmed diagnosis of hypertension in pregnancy were also excluded from the analysis, as their indication could not be determined or adjusted for in our analysis model.

### Randomisation and masking

As this was an observational study emulating a target trial, no randomisation occurred. However, we balanced covariates between groups using inverse probability-weighting (IPW). IPW is a technique whereby each participant is assigned a score based on their likelihood of receiving either treatment, relative to the presence of confounding factors.^27^ When these scores are applied to the population, covariates can be balanced across the exposure groups, as they would be in a randomised trial.^27^ This study emulates an open-label trial as blinding is not possible using retrospective data.

### Outcome measures

Our co-primary outcomes were composites of maternal and neonatal severe adverse outcomes. The maternal composite outcome comprised: maternal mortality (all-cause); eclampsia; haemolysis, elevated liver enzymes, low platelet count (HELLP) syndrome; stroke; cortical blindness; retinal detachment; pulmonary oedema; placental abruption; renal failure requiring dialysis; and organ failure requiring extracorporeal membrane oxygenation (ECMO). These outcomes were selected to represent the most severe sequelae of hypertensive disorders of pregnancy for women.^28^, ^29^, ^30^

The neonatal composite outcome comprised: stillbirth ≥20 weeks’ gestation; neonatal death ≤28 days postpartum; birthweight ≤3rd centile; cardiac arrest or heart failure; hypoxic-ischemic encephalopathy; intracranial haemorrhage; invasive resuscitation (intubation or mechanical ventilation); non-invasive resuscitation; necrotizing enterocolitis; neonatal asphyxia; and neonatal seizures. These outcomes were again selected as they represent the most severe sequalae of maternal hypertensive disorders for neonates: fetal growth restriction and preterm birth.^29^, ^30^, ^31^

Secondary outcomes included requirement for additional antihypertensives, disease progression (gestational hypertension to preeclampsia post-randomisation), iatrogenic preterm birth (induction of labour or caesarean section not in labour), major congenital abnormality (EUROCAT criteria),^32^ neonatal hypoglycaemia, and birthweight ≤3rd centile (Hadlock).^33^

All outcomes were identified from: the Victorian Perinatal Data Collection (VPDC); the Victorian Emergency Minimum Dataset (VEMD) and Victorian Admitted Episodes Dataset (VAED), which together include information on all public and private hospital admissions and emergency department presentations across Victoria, including diagnoses and procedures; and the National Death Index (NDI), a registry of all deaths in Australia. Diagnostic codes were identified and validated from the International Statistical Classification of Diseases and Related Health Problems, 10th Revision, Australian Modification (ICD-10-AM). Procedure codes were identified and validated from the Australian Classification of Health Interventions (ACHI), 8th edition. A full list of outcome definitions is included in Supplement 2, Table S9.

### Covariates

The multidisciplinary authorship team decided *a priori* which covariates should be considered for inclusion in the analytical model. We used direct acyclic graphs (DAGs) to describe the direction and structure of potential causal relationships between exposure, covariates, and outcomes, and to identify those required in the selection and outcome models (Supplement 1).

The covariates selected for inclusion in our analytical models were: indication for medication use (i.e., type of hypertensive disorder at time of first dispensation—gestational hypertension, chronic hypertension, or preeclampsia); maternal age at birth; maternal body mass index (BMI); conception via assisted reproductive technology; maternal pre-existing comorbidities; gestational diabetes; smoking during pregnancy; socioeconomic status; plurality; parity; and mode of birth (for neonatal outcomes only). Neonatal analyses were not adjusted for gestational age at birth due to its role as a mediator (aside from birthweight centile for gestational age).^34^, ^35^, ^36^

### Missing data

The proportions of missing data are described in Table 2. Data were only missing for covariates and outcomes; no exposure data were missing. Data that were missing at random or completely at random were imputed, even if missingness was low.^37^^,^^38^ Imputed covariates included maternal body mass index (BMI), assisted reproductive technology conception, socioeconomic status, smoking status, and neonatal birthweight.

**Table 2:** Baseline characteristics of cohorts.

Maternal characteristics	Labetalol (N = 6745)	Nifedipine (N = 671)	p value
Maternal age, mean (SD)	33.1 (5.6)	34.0 (6.0)	<0.001
Missing (%)	0 (0)	0 (0)	
Maternal age ≥40 years (%)	736 (10.9)	109 (16.2)	<0.001
Parity (%)			0.011
Nulliparous	3185 (47.2)	283 (42.2)	
Multiparous	3560 (52.8)	388 (57.8)	
Maternal clustering (%)			0.194
1	5550 (82.3)	538 (80.2)	
2	1064 (15.8)	114 (17.0)	
3+	131 (1.9)	19 (2.8)	
Diagnosis at initiation of antihypertensive treatment (%)			<0.001
Chronic hypertension	1580 (23.4)	174 (25.9)	
Gestational hypertension	2846 (42.2)	228 (34.0)	
Preeclampsia	2319 (34.4)	270 (40.2)	
Marital status (%)			0.301
Single	875 (13.0)	81 (12.1)	
Married/de facto	5693 (84.4)	574 (85.5)	
Separated/divorced/missing	177 (2.6)	16 (2.4)	
Gestation at first exposure (%)			<0.001
11 + 0 to 19 + 6 weeks	1409 (20.9)	168 (25.0)	
20 + 0 to 27 + 6 weeks	971 (14.4)	145 (21.6)	
28 + 0 to 36 + 6 weeks	4365 (64.7)	358 (53.4)	
Assisted reproductive technology conception (%)	533 (7.9)	92 (13.7)	<0.001
Missing	21 (0.3)	<6 (<0.5)	
Body mass index (mean, SD)	31.8 (7.5)	30.7 (7.8)	0.001
Missing	304 (4.5)	51 (7.6)	<0.001
Body mass index ≥30 (%)	3456 (51.2)	301 (44.9)	0.013
Maternal comorbidities (%)			
Type 1 or 2 diabetes mellitus	193 (2.9)	30 (4.5)	0.020
Renal disease	102 (1.5)	20 (3.0)	0.005
Autoimmune disease	91 (1.4)	21 (3.1)	<0.001
Smoking in pregnancy (%)	1103 (16.4)	112 (16.7)	0.609
Missing	56 (0.8)	8 (1.2)	
Socioeconomic Index for Areas Quintile (%)			0.001
1 (Most disadvantaged)	1084 (16.1)	90 (13.4)	
2	1102 (16.3)	106 (15.8)	
3	1756 (26.0)	146 (21.8)	
4	1632 (24.2)	172 (25.6)	
5 (Least disadvantaged) or missing	1171 (17.4)	157 (23.4)	
Plurality (%)			0.028
Singleton	6634 (98.4)	653 (97.3)	
Twins or higher order birth	111 (1.7)	18 (2.7)	
Missing	0 (0)	0 (0)	
Gestational diabetes mellitus (%)	1532 (22.7)	143 (21.3)	0.399
Missing	0 (0)	0 (0)	
Year of delivery (%)			<0.001
2009	109 (85.8)	18 (14.2)	
2010	130 (89.0)	16 (11.0)	
2011	137 (91.3)	13 (8.7)	
2012	387 (88.4)	51 (11.6)	
2013	572 (87.1)	85 (12.9)	
2014	617 (90.5)	65 (9.5)	
2015	681 (90.4)	72 (9.6)	
2016	679 (89.9)	76 (10.1)	
2017	759 (91.3)	72 (8.7)	
2018	799 (92.2)	68 (7.8)	
2019	892 (92.9)	68 (7.1)	
2020	985 (93.5)	68 (6.5)	
Missing	0 (0)	0 (0)	

Neonatal characteristics	Labetalol (N = 6855)	Nifedipine (N = 690)	p value
Sex at birth (%)			0.162
Female	3217 (46.9)	346 (50.2)	
Male or missing	3638 (53.1)	344 (49.8)	
Mode of birth (%)			0.014
Unassisted vaginal birth	2098 (30.6)	192 (27.8)	
Operative vaginal birth (forceps or ventouse)	707 (10.3)	54 (7.8)	
Planned caesarean section	1788 (26.1)	212 (30.7)	
Unplanned/emergency caesarean section	2262 (33.0)	232 (33.6)	
Missing	0 (0)	0 (0)	
Gestational age at birth (mean, SD), weeks	37.2 (2.5)	36.5 (3.1)	<0.001
Missing	0 (0)	0 (0)	
Birthweight (mean, SD), grams	2924.3 (742.1)	2845.8 (831.1)	0.006
Missing	8 (0.1)	<6 (<0.5)	

All cells with <6 individuals have been censored to maintain anonymity.

p-Values calculated from Chi-square test (categorical variables) or Student's t-test (continuous variables).

SD = standard deviation.

Bootstrapping was performed as per the von Hippel method.^38^ In brief, 1000 bootstrapped samples (B = 1000) were obtained from the source data, and two imputed datasets (M = 2) were obtained for each bootstrapped sample (n = 2000 analysis datasets). A full description of our approach to missing data, imputation, and bootstrapping is provided in Supplement 1 and has been previously reported by our team.^39^, ^40^, ^41^

### Statistical analysis and causal contrasts

Statistical analyses were performed between December 2024 and November 2025. The distribution of maternal and neonatal characteristics was described using mean (SD), and number (%), according to the type and distribution of the data (Table 2). Chi-squared tests and Student's t-tests were conducted for categorical variables and continuous variables, respectively, based on advice from McCrum-Gardner.^42^

The average treatment effect (ATE) used in this study is based on the potential outcomes framework, detailed in the Supplement 1.^43^^,^^44^ The estimands for the primary outcomes are presented as the ATE and 95% confidence intervals (95% CI) and defined as the between-treatment risk difference (RD) and relative risk (RR).

The primary estimator used a doubly robust inverse probability-weighted (IPW, described above) selection model with a standard regression adjustment (RA) outcome model to calculate an individual's likelihood of both exposure and outcome. For each covariate, the standardised mean difference between exposure arms was calculated to assess if balance between weighted pseudo-populations was achieved (Supplement 1). This is a standard diagnostic technique which allows for quantitative comparison of covariate balance in an inverse probability-weighted model.^45^ A standardised mean difference <0.1 indicates that balance has been achieved.^45^^,^^46^ Sufficient balance was achieved for all measured covariates, supporting unbiased causal estimates (Supplement 1).

All estimators used the same bootstrapped, multiply imputed datasets (n = 2000). Maternal clustering was accounted for in the analysis models via standard error adjustment using a unique maternal identifier.

As this study used retrospective data, no blinding or randomisation occurred; although study covariates were balanced using inverse probability-weighting. Our sample size was determined based on available data (i.e., all eligible women were included). A power calculation was performed using a Chi-squared test and it was determined that we were adequately powered (80%) to detect a difference of ≥3.0% in the maternal primary outcome and ≥5.6% for the neonatal primary outcome. Inclusion and exclusion criteria are described above and in detail within Supplement 1. Sensitivity and post-hoc analyses are described below.

Statistical analysis was performed using Stata statistical software, version 19 (StataCorp, LLC),^47^ including the *teffects* suite.

### Ad hoc sensitivity analyses

Sensitivity analyses for the primary outcomes included (1) a complete case analysis; and (2) a per-protocol analysis (excluding cases of crossover). We also completed subgroup analyses by: (1) indication for treatment (chronic or gestational hypertension versus preeclampsia); (2) gestational age at birth (≤34 weeks versus > 34 weeks' gestation) for neonatal outcomes; and (3) timing of first prescription (11 + 0 to 19 + 6 weeks versus 20 + 0 to 27 + 6 weeks versus 28 + 0 to 36 + 6 weeks’ gestation).

### Post-hoc sensitivity analyses

Several post-hoc sensitivity analyses were conducted following consultation with consumers and peer review. First, we conducted a sensitivity analysis adjusted for year of delivery based on consumer feedback, to account for changes in prescribing patterns over the course of the study. Next, we conducted a sensitivity analysis excluding women with asthma (a potential contraindication for labetalol) and using only the first pregnancy episode per included woman, based on reviewer feedback. Finally, we conducted a sensitivity analysis using propensity score matching instead of inverse probability-weighted regression adjustment, again based on reviewer feedback.

We also calculated E-values to quantify the potential effect of unmeasured confounders for our primary and secondary outcomes.^48^^,^^49^ The E-value estimates the association that an unmeasured confounder would need to have with both treatment assignment (exposure) and outcome to fully explain away the associations observed.^48^^,^^49^

### Ethics and governance

Ethical approval for this project was provided by the Mercy Health Human Research Ethics Committee (HREC 2020-048) and Australian Institute of Health and Welfare (AIHW EO2021-1-1175). Given the retrospective and deidentified nature of this study, the requirement for individual participant informed consent was waived by both the Mercy HREC and AIHW. Data were linked by the Centre for Victorian Data Linkage and AIHW. All analyses were conducted within the Secure Unified Research Environment (SURE), hosted by the Sax Institute, as mandated by AIHW.

### Consumer involvement

The Mercy Perinatal Lived Experience (MaPLE) consumer group was consulted during the preparation of this manuscript, helping to interpret our findings and guide any further analyses.

### Role of the funding source

The funder of the study had no role in study design, data collection, data interpretation, or writing of the report.

## Results

From 2009 to 2020, 880,185 pregnancies were captured in our Victorian dataset. Of these, 7416 (0.8%) met inclusion criteria and were included in the final analysis cohort (Fig. 1). 6745 (91.0%) women were first prescribed labetalol and 671 (9.0%) were first prescribed nifedipine. 1729 (23.3%) women were already taking antihypertensives prior to randomisation, while the remaining 5688 (76.7%) were new users of antihypertensive medications.

Compared with women who received labetalol as first-line treatment, those who received nifedipine were slightly older, had a lower BMI, and were more likely to have multiple pregnancies, comorbidities, be of a higher socioeconomic background, and have conceived via assisted reproductive technology. Women in the nifedipine group were also more likely to have an indication of preeclampsia or chronic hypertension, rather than gestational hypertension. Infants in the nifedipine group were more likely to be born via planned caesarean section and were on average of an earlier gestational age and lower birthweight (Table 2).

### Primary composite maternal outcome

For the composite maternal outcome, nifedipine was associated with a 42% unadjusted increased risk when compared with labetalol (6.6% versus 9.4%; RR 1.42, 95% CI 1.10, 1.82). After adjusting via inverse probability-weighted regression adjustment (IPWRA), women in the nifedipine group were 33% more likely to experience the composite maternal outcome, compared with those in the labetalol group (aRR 1.33, 95% CI 1.07, 1.64) (Table 3). This was largely driven by increased diagnoses of eclampsia (1.6% versus 3.0%), HELLP syndrome (3.0% versus 4.3%), and renal failure requiring dialysis (1.0% versus 1.5%) in the nifedipine group (Table 4).

**Table 3:** Primary maternal and neonatal outcomes.

	Observed proportions		Unadjusted effect estimates		Adjusted effect estimates^a^	
	Labetalol (%)	Nifedipine (%)	Risk difference (95% CI) (%)	Risk ratio (95% CI)	Risk difference (95% CI) (%)	Risk ratio (95% CI)
Composite maternal outcome						
All hypertensive disorders	447 (6.6)	63 (9.4)	2.76 (0.48, 5.05)	1.42 (1.10, 1.82)	2.25 (0.37, 4.13)	1.33 (1.07, 1.64)
Preeclampsia^b^	273 (11.8)	43 (15.9)	4.14 (−0.40, 8.71)	1.35 (1.01, 1.82)	3.96 (2.63, 5.28)	1.32 (1.21, 1.45)
Gestational or chronic hypertension^b^	174 (3.9)	20 (5.0)	1.06 (−1.15, 3.26)	1.27 (0.81, 1.99)	1.58 (−0.58, 3.73)	1.39 (0.91, 2.11)
Composite neonatal outcome						
All hypertensive disorders	2947 (43.0)	318 (46.1)	3.10 (−0.80, 7.00)	1.07 (0.98, 1.17)	−1.25 (−5.15, 2.64)	0.97 (0.89, 1.06)
Preeclampsia^b^	1261 (53.2)	159 (55.6)	2.39 (−3.71, 8.49)	1.04 (0.94, 1.17)	−1.94 (−5.80, 1.91)	0.96 (0.89, 1.04)
Gestational or chronic hypertension^b^	1686 (37.6)	159 (39.4)	1.76 (−3.21, 6.73)	1.05 (0.92, 1.19)	−1.09 (−6.70, 4.52)	0.97 (0.83, 1.13)

CI = confidence interval.

**Maternal composite outcome:** Maternal mortality (all-cause); eclampsia; HELLP syndrome; stroke; cortical blindness; retinal detachment; pulmonary oedema; placental abruption; liver rupture; renal failure requiring dialysis; organ failure requiring extracorporeal membrane oxygenation.

**Neonatal composite outcome:** Stillbirth (≥20 weeks' gestation); neonatal death (≤28 days post-birth); neonatal seizures; necrotizing enterocolitis; hypoxic-ischemic encephalopathy; birthweight ≤3rd centile (Hadlock); invasive resuscitation; non-invasive resuscitation; bacterial sepsis; intracranial haemorrhage; neonatal asphyxia; heart failure or cardiac arrest.

a All models adjusted for the following covariates: type of hypertension; maternal age at birth; maternal BMI; conception via artificial reproductive technology; maternal comorbidities (type 1 or 2 diabetes mellitus, renal disease, autoimmune disease); gestational diabetes mellitus; smoking during pregnancy; socioeconomic status; plurality; and parity. Neonatal models also adjusted for mode of birth.

b Diagnosis at treatment initiation (randomisation).

**Table 4:** Components of maternal and neonatal primary composite outcomes.

Component	Labetalol (%)	Nifedipine (%)
Maternal primary composite outcome	447 (6.6)	63 (9.4)
Maternal mortality (all-cause)	0 (0)	0 (0)
Eclampsia	105 (1.6)	20 (3.0)
Haemolysis, elevated liver enzymes, low platelet count (HELLP) syndrome	200 (3.0)	29 (4.3)
Stroke	10 (0.2)	0 (0)
Cortical blindness	0 (0)	0 (0)
Retinal detachment	<6 (<0.5)	0 (0)
Placental abruption	99 (1.5)	8 (1.2)
Pulmonary oedema	7 (0.1)	0 (0)
Liver failure	0 (0)	0 (0)
Renal failure requiring dialysis	69 (1.0)	10 (1.5)
Extracorporeal membrane oxygenation	0 (0)	0 (0)
Neonatal primary composite outcome	2947 (43.0)	318 (46.1)
Stillbirth ≥20 weeks' gestation	59 (0.9)	12 (1.7)
Neonatal death ≤28 days post-birth	12 (0.2)	<6 (<0.5)
Neonatal seizures	20 (0.3)	0 (0)
Necrotizing enterocolitis	33 (0.5)	<6 (<0.5)
Hypoxic-ischemic encephalopathy	8 (0.1)	<6 (<0.5)
Birthweight ≤3rd centile (Hadlock)	772 (10.5)	75 (10.9)
Invasive resuscitation (intubation or mechanical ventilation)	202 (3.0)	38 (5.5)
Non-invasive resuscitation	2400 (35.0)	259 (37.5)
Bacterial sepsis	533 (7.8)	53 (7.7)
Intracranial haemorrhage	56 (0.8)	<6 (0.7)
Neonatal asphyxia	47 (0.7)	7 (1.0)
Heart failure or cardiac arrest	<6 (<0.5)4	<6 (<0.5)

All cells with <6 individuals have been suppressed to maintain anonymity.

After stratifying by type of hypertensive disorder at first prescription of either drug (preeclampsia, chronic or gestational hypertension), only women with preeclampsia remained at significantly increased risk of the composite maternal outcome (11.8% versus 15.9%; aRR 1.32, 95% CI 1.21, 1.45). The risk was not significant for women with chronic or gestational hypertension at commencement of medication (3.9% versus 5.0%; aRR 1.39, 95% CI 0.91, 2.11).

### Primary composite neonatal outcome

The absolute incidence of the composite neonatal outcome was high for both the labetalol (43.0%) and nifedipine (46.1%) groups. In unadjusted analyses, there was no association between either medication and the composite neonatal outcome (RR 1.07, 95% CI 0.98, 1.17). Importantly, after adjusting via IPWRA, we found no difference in the risk of the composite neonatal outcome between either antihypertensive agent (aRR 0.97, 95% CI 0.89, 1.06) (Table 3). There was also no difference between groups when stratifying by indication (preeclampsia: aRR 0.96, 95% CI 0.89, 1.04; chronic or gestational hypertension: aRR 0.97, 95% CI 0.83, 1.13) or gestational age at birth (≤34 weeks' gestation: aRR 1.02, 95% CI 1.00, 1.05; >34 weeks’ gestation: aRR 0.90, 95% CI 0.80, 1.02) (Supplement 2, Table S4).

### Secondary maternal outcomes

Women in the nifedipine group were 2.35 times more likely to require additional antihypertensives to manage blood pressure (i.e., additionally prescribed labetalol and/or methyldopa), compared with those in the labetalol group (aRR 2.35, 95% CI 2.20, 2.51) However, there was no difference between groups in the risk of progression from gestational hypertension to preeclampsia (aRR 1.01, 95% CI 0.82, 1.24) (Table 5).

**Table 5:** Secondary maternal and neonatal outcomes.

	Observed proportions		Unadjusted effect estimates		Adjusted effect estimates^a^	
	Labetalol (%)	Nifedipine (%)	Risk difference (95% CI) (%)	Risk ratio (95% CI)	Risk difference (95% CI) (%)	Risk ratio (95% CI)
Secondary maternal outcomes						
Requirement for additional antihypertensives	1284 (19.0)	316 (47.1)	28.1 (24.2, 31.9)	2.48 (2.25, 2.72)	26.6 (23.6, 29.5)	2.35 (2.20, 2.51)
Disease progression (gestational hypertension to preeclampsia)	905 (31.3)	65 (28.3)	−3.00 (−9.06, 3.06)	0.90 (0.73, 1.12)	0.38 (−6.08, 6.83)	1.01 (0.82, 1.24)
Secondary neonatal outcomes						
Iatrogenic preterm birth						
*<37 weeks' gestation*	1832 (26.7)	243 (35.2)	8.49 (4.78, 12.21)	1.32 (1.18, 1.47)	7.01 (4.10, 9.92)	1.26 (1.15, 1.38)
*<34 weeks' gestation*	538 (7.9)	88 (12.8)	4.89 (2.32, 7.46)	1.62 (1.31, 2.00)	4.19 (3.53, 4.85)	1.52 (1.44, 1.61)
*<32 weeks' gestation*	269 (3.9)	49 (7.1)	3.18 (1.21, 5.15)	1.81 (1.35, 2.43)	2.48 (1.37, 3.59)	1.62 (1.36, 1.93)
*<28 weeks' gestation*	87 (1.3)	19 (2.8)	1.48 (0.24, 2.73)	2.17 (1.33, 3.54)	1.77 (1.39, 2.15)	2.39 (2.04, 2.79)
Major congenital abnormality	423 (6.2)	51 (7.4)	1.22 (−0.82, 3.25)	1.20 (0.91, 1.58)	0.39 (−1.60, 2.37)	1.05 (0.79, 1.40)
Neonatal hypoglycaemia	1635 (23.9)	153 (22.2)	−1.68 (−4.94, 1.58)	0.93 (0.80, 1.08)	−5.07 (−6.07, −4.07)	0.79 (0.75, 0.83)
Birthweight ≤3rd centile	722 (10.5)	75 (10.9)	0.36 (−2.08, 2.80)	1.03 (0.83, 1.29)	−0.60 (−2.10, 0.90)	0.94 (0.81, 1.09)

CI = confidence interval.

**Maternal composite outcome:** Maternal mortality (all-cause); eclampsia; HELLP syndrome; stroke; cortical blindness; retinal detachment; pulmonary oedema; placental abruption; liver rupture; renal failure requiring dialysis; organ failure requiring extracorporeal membrane oxygenation.

**Neonatal composite outcome:** Stillbirth (≥20 weeks' gestation); neonatal death (≤28 days post-birth); neonatal seizures; necrotizing enterocolitis; hypoxic-ischemic encephalopathy; birthweight ≤3rd centile (Hadlock); invasive resuscitation; non-invasive resuscitation; bacterial sepsis; intracranial hemorrhage; neonatal asphyxia; heart failure or cardiac arrest.

a All models adjusted for the following covariates: type of hypertension; maternal age at birth; maternal BMI; conception via artificial reproductive technology; maternal comorbidities (type 1 or 2 diabetes mellitus, renal disease, autoimmune disease); gestational diabetes mellitus; smoking during pregnancy; socioeconomic status; plurality; and parity. Neonatal models also adjusted for mode of birth.

### Secondary neonatal outcomes

Compared with oral labetalol, nifedipine was associated with a 26% increased risk of iatrogenic preterm birth (<37 weeks' gestation) (aRR 1.26, 95% CI 1.15, 1.38). This risk increased in a stepwise manner for iatrogenic preterm birth <34 weeks' gestation (aRR 1.52, 95% CI 1.44, 1.61); <32 weeks' gestation (aRR 1.62, 95% CI 1.36, 1.93); and <28 weeks’ gestation (aRR 2.04, 95% CI 2.04, 2.79) (Table 5).

There was no difference in the risk of birthweight ≤3rd centile between groups (10.5% versus 10.9%; aRR 0.94, 95% CI 0.81, 1.09) or major congenital abnormality (6.2% versus 7.4%; aRR 1.05, 95% CI 0.79, 1.40). However, nifedipine was associated with a 21% decreased risk of neonatal hypoglycemia (23.9% versus 22.2%; aRR 0.79, 95% CI 0.75, 0.83) (Table 5).

### Sensitivity analyses

Our composite maternal outcome remained similar in our complete-case and per-protocol sensitivity analyses. Our composite neonatal outcome also remained unchanged in all sensitivity analyses (Tables S1–S4, S6–S8, S10).

Next, we stratified our analysis by timing of exposure (11 + 0 to 19 + 6 weeks' gestation, 20 + 0 to 27 + 6 weeks' gestation, and 28 + 0 to 36 + 6 weeks' gestation). For the maternal primary outcome, we found no difference between nifedipine and labetalol for those commencing early in pregnancy (11 + 0 to 19 + 6 weeks' gestation; aRR 0.95, 95% CI 0.65, 1.37). Conversely, for those commencing in mid-gestation (20 + 0 to 27 + 6 weeks' gestation), nifedipine was associated with a 57% increased risk of the composite maternal outcome (aRR 1.57, 95% CI 1.44, 1.71), and 19% increased risk for those commencing in the third trimester (28 + 0 to 36 + 6 weeks’ gestation; aRR 1.19, 95% CI 1.07, 1.32), when compared with labetalol. Our composite neonatal outcome again remained unchanged (Supplement 2, Table S3).

Finally, we calculated E-values for all statistically significant primary and secondary maternal and neonatal outcomes to estimate the effect of unmeasured confounding (Supplement 2, Table S10).^48^^,^^49^ For the primary maternal outcome, the calculated E-value of the adjusted risk ratio (aRR 1.33, 95% CI 1.07, 1.64) was 1.99 (1.34). This means that an unmeasured confounder associated with both treatment assignment and the maternal primary outcome by a magnitude equivalent to or greater than a risk ratio of 1.99 could nullify the observed association, but a confounder with a lower risk ratio could not. Similarly, the lower bound of the confidence interval could be explained away by an unmeasured confounder associated with both treatment assignment and the maternal primary outcome with a risk ratio equal to or greater than 1.34. The calculated E-values for all statistically significant outcomes suggest that our conclusions are overall robust to unmeasured confounding bias (Supplement 2, Table S10).

### Consumer feedback and post-hoc analyses

Following consultation with our consumer advisory group, three key themes were identified: 1) the potential impact on our results of changing prescribing practices over the course of the study period; 2) the importance of our study inclusion and exclusion criteria; and 3) the critical role of future communication of our findings to consumers.

Based on this feedback, we examined the pattern of prescribing throughout the course of our study (2009–2020) and found there were changes, with nifedipine becoming less commonly prescribed over this time (Table 2). To address this, we conducted a *post hoc* analysis of our primary outcomes, adjusting additionally for year of birth. We found no significant differences in our primary outcomes (Supplement 2, Table S5).

Our consumer group highlighted the importance of emphasising that this study was conducted in a very high-risk group of women (hypertension requiring pharmacological treatment) and examined relatively rare outcomes. It is important to note that these findings may not be generalisable to low-risk populations.

Finally, our consumer advisors were determined to highlight that while it appears labetalol is associated with more favourable patient outcomes compared with nifedipine, both medications are associated with significantly improved outcomes for mothers and babies compared with untreated hypertension.^50^ We will continue working with consumer groups to develop plain language summaries to ensure our findings are accessible to those directly affected by this data.

## Discussion

Using a causal framework based on target trial emulation, we assessed the effect of oral labetalol and nifedipine on adverse maternal and neonatal outcomes in the setting of hypertensive disorders of pregnancy before 37 weeks’ gestation.

We demonstrated a significant maternal benefit with oral labetalol (2.25% absolute risk reduction of severe morbidity or mortality), with no evidence of associated fetal harm. This benefit was most significant among women with preeclampsia (3.96% absolute risk reduction). Labetalol was also associated with a significantly reduced risk of iatrogenic preterm birth, even at extremely preterm gestations.

The mechanisms behind these findings are unclear. Previous studies have shown little difference in the risk of adverse outcomes between pregnancies treated with labetalol and nifedipine.^10^, ^11^, ^12^, ^13^^,^^51^, ^52^, ^53^, ^54^, ^55^, ^56^, ^57^ However, these studies have largely been limited to the setting of their use in a hypertensive crisis or were underpowered to assess serious morbidity and mortality as primary outcomes. A 2025 target trial emulation examined oral labetalol versus nifedipine for the treatment of chronic hypertension in pregnancy and showed no difference any examined outcomes, including the risk of developing severe preeclampsia or eclampsia.^57^ While their sample size was similar to the present study (N = 6724), this study included only women with chronic hypertension, and examined a composite of maternal and neonatal outcomes (severe preeclampsia/eclampsia, medically indicated preterm birth, placental abruption, and stillbirth), finding no significant difference between groups.^57^ Likewise, we saw no difference in the risk of adverse maternal and neonatal outcomes among women with chronic or gestational hypertension.

Conversely, A 2022 network meta-analysis showed 37% less proteinuria among pregnant women treated with labetalol, compared with calcium channel blockers.^10^ Similar results were seen in a 2017 randomised trial, which showed increased proteinuria across gestation for nifedipine-treated women with chronic hypertension, as compared with oral labetalol.^11^ Increased proteinuria with nifedipine treatment may suggest a disease modifying effect beyond blood pressure control if labetalol is given instead, but this is speculative.

In the non-pregnant population, nifedipine has been shown to be less effective than other antihypertensives (including beta-blockers) in preventing proteinuria and renal damage.^3^^,^^10^^,^^58^, ^59^, ^60^, ^61^, ^62^, ^63^, ^64^, ^65^, ^66^ This suggests that labetalol may be more effective in preserving renal function than nifedipine. Indeed, we observed reduced rates of eclampsia, HELLP syndrome, and renal failure requiring dialysis in the labetalol group, suggesting possible vascular and renal protection. Increased proteinuria is also associated with poorer neonatal outcomes, including preterm birth, intrauterine fetal death, and fetal distress.^67^ Given the already compromised renal function of women with preeclampsia, these trends warrant further investigation.^68^

We also observed that oral labetalol was associated with a decreased risk of iatrogenic preterm birth. This contrasts the 2025 target emulation conducted by Leonard et al. which found no difference in the risk of medically indicated preterm birth.^57^ However, this study included only chronic hypertension and did not examine preeclamptic or gestational hypertension pregnancies, thus making it difficult to compare findings.^57^ Most iatrogenic preterm births in our cohort were due to maternal hypertension—suggesting poorer blood pressure control in the nifedipine group rather than a direct effect of nifedipine on length of gestation. This premise is further supported by the increased requirement for additional antihypertensives that occurred among those using nifedipine, compared with labetalol.

Studies have suggested that nifedipine can more quickly control maternal blood pressure in the setting of hypertensive crises.^12^^,^^51^, ^52^, ^53^, ^54^, ^55^, ^56^ These studies included women with both gestational hypertension and preeclampsia, but did not stratify outcomes. Conversely, a 2020 study showed that intravenous labetalol was more effective than nifedipine in controlling blood pressure among a group of 50 preeclamptic women.^69^ In this study, nifedipine was associated with an increased risk of preterm induction of labour, NICU admission, low birthweight, and low 5-min Apgar scores.^69^ Additionally, a 2019 randomised trial showed that, while oral nifedipine and labetalol were equally effective, oral nifedipine was associated with an increased risk of NICU admission among women with severe hypertension.^9^ Our findings of an increased risk of maternal morbidity and iatrogenic preterm birth among the nifedipine group seem to suggest it is a less effective antihypertensive than labetalol; however, these trends require further investigation.

A strength of this study is the size of our cohort and the performing of target trial emulation. By using population-level data, we were powered to assess severe maternal and neonatal adverse events as primary outcomes. To our knowledge, this is the first sufficiently powered study to do so. Utilizing target trial emulation, we have avoided biases often associated with observational studies.^22^ We have further reduced bias by using contemporary statistical tools, such as multiple imputation and bootstrapping.^37^^,^^38^

Our study has important limitations. We only had access to outpatient prescriptions. It is possible participants received additional antihypertensives from the hospital which would have been uncaptured on our database. However, given the focus of this study is antihypertensive use in the non-emergency setting, we anticipate acute use of antihypertensives of relatively short durations would not significantly influence our findings. We also did not have access to neonatal intensive care unit (NICU) admissions data. We instead selected severe neonatal morbidities that were likely to result in NICU admission for our neonatal composite outcome. This provides a robust picture of the most severe outcomes of maternal hypertension and prematurity for the neonate; however, we may have missed less severe morbidity, which can still lead to NICU admission and associated burden.

Women with pre-existing chronic hypertension were included to better emulate the Giant PANDA trial and make our findings more generalisable to the general maternity population.^13^ Around 1% of women giving birth in Australia will have chronic hypertension, representing 13% of all hypertensive disorders of pregnancy.^70^ It is therefore imperative that we understand which antihypertensives work best for this population, in addition to women with new-onset hypertension, to ensure they are receiving the best possible standard of care.

We combined women with chronic and gestational hypertension in our stratified analyses due to a small number of women in the chronic hypertension group experiencing the primary maternal outcome (n < 5 in one of the medication arms). We elected to combine women with chronic and gestational hypertension due to their similar likelihood of developing severe maternal outcomes (relative to women with preeclampsia) and similar management strategies in Australia.^24^^,^^71^ Future research investigating these groups separately may demonstrate different results.

Our study also pools data from an eleven-year period. It is possible that changes in prescribing practices during this time may have influenced our findings. However, when adjusting for year of birth, we saw little difference in our primary findings (Supplement 2, Table S5). Guidelines surrounding antihypertensive therapy in Australia remained largely unchanged over the study period—both the 2008 and 2014 Society for Obstetric Medicine Australia and New Zealand (SOMANZ) guidelines recommend commencing treatment for all women with blood pressure ≥160/100–110 mmHg, with local practice to dictate treatment for women with lower blood pressure.^72^^,^^73^ Both also have similar definitions of gestational hypertension, chronic hypertension, and preeclampsia, and the same indications for delivery in both hypertensive and preeclamptic women.^72^^,^^73^ The 2014 guideline recommends aiming for a systolic blood pressure of 130–140 mmHg and a diastolic blood pressure of 80–90 mmHg, while the 2008 guideline had no specific targets.^72^^,^^73^ These same guidelines indicate that nifedipine and labetalol are considered suitable for stable (non-severe) hypertension in pregnancy.^72^^,^^73^

Our data showed a strong selection of labetalol over nifedipine across the study period, suggesting possible selection bias. We were unable to identify any clinical explanation for this discrepancy, as both labetalol and nifedipine are recommended as first-line treatments in Australia, with no guidance to suggest the selection of one over the other.^24^ While there may be clinician bias towards labetalol, we are unable to ascertain the reasons for this from our data. However, we were able to eliminate potential selection bias by performing inverse probability-weighting to balance the groups across key covariates.^27^^,^^74^ The success of this approach is shown in Supplement 1 and outlined in Methods. In addition, we performed a propensity score-matched sensitivity analysis which yielded similar results to our primary analyses. Further, our population consisted only of women with a confirmed diagnosis of hypertension, which is already a highly selected group, with similar baseline characteristics when compared with the general pregnant population.^75^

This study utilised bootstrapping and multiple imputation to handle missing data and calculate standard errors.^38^ While this is a robust method, there are some important limitations to consider. The quality of our bootstrapped datasets depends on the correctness of our imputation model; it is possible that this may be misspecified, and therefore lead to inaccurate results. To overcome this, we employed maximum likelihood estimation (MLE) to increase convergence, carefully selected covariates for both the imputation and analysis models (by using direct acyclic graphs), and performed recommended diagnostic tests on imputed datasets.^76^ Additionally, we performed a complete case sensitivity analysis, which produced similar results to our imputed data (Supplement 2, Table S1).

Whilst our data were not truly randomised, we used a doubly robust inverse probability-weighted regression analysis to balance baseline characteristics, as randomisation would. The success of this model is demonstrated by achieving adequate covariate balance between groups (Supplement 1). However, it is possible that unmeasured residual confounding or model misspecification could have led to bias in estimating the average treatment effect.^77^ In particular, we did not have access to maternal blood pressure or laboratory measurements (for example, serum creatinine or proteinuria). Given that antihypertensive treatment should be titrated to blood pressure, it is possible that including baseline blood pressure in our analysis model may influence our findings.^78^ However, our calculated E-values suggest that overall our results are robust to the effect of unmeasured residual confounding, suggesting that including additional covariates would be unlikely to sway our conclusions (Supplement 2, Table S10).

In this statewide, population-based cohort study using a causal inference framework, we observed a significant maternal benefit associated with labetalol use, compared with nifedipine, among women with hypertensive disorders of pregnancy before 37 weeks’ gestation. We also showed no significant difference in neonatal morbidity and mortality between either drug; however, oral labetalol use was associated with a reduced risk of iatrogenic preterm birth. These findings suggest that oral labetalol may offer treatment benefit as the preferred first-line antihypertensive among pregnant women, particularly those with preeclampsia.

## Contributors

Jessica A Atkinson: Conceptualisation; Methodology; Validation; Formal Analysis; Investigation; Writing—Original Work; Visualisation; Funding Acquisition.

Anthea C Lindquist: Conceptualisation; Methodology; Validation; Writing—Review & Editing; Supervision; Funding Acquisition.

Stephen Tong: Conceptualisation; Methodology; Writing—Review & Editing; Supervision.

Richard J Hiscock: Methodology; Formal Analysis; Writing—Review & Editing.

Anna Forsythe: Data Curation; Writing—Review & Editing; Project Administration.

Hannah G Gordon: Methodology; Data Curation; Writing—Review & Editing.

Susan P Walker: Methodology; Writing—Review & Editing.

Su Jen Chua: Methodology; Writing—Review & Editing.

Catherine Cluver: Methodology; Writing—Review & Editing.

Jenny Myers: Methodology; Writing—Review & Editing.

Roxanne M Hastie: Conceptualisation; Methodology; Validation; Writing—Review & Editing; Supervision; Funding Acquisition.

JAA and ACL contributed equally to this work and are the co-first authors. JAA, RMH, and ACL have accessed and verified the underlying study data and take full responsibility for the integrity of the data, the accuracy of the data analysis, and for the decision to submit for publication. They are the guarantors. JAA attests that all listed authors meet authorship criteria and that no others meeting criteria have been omitted. All authors read and approved of the final version of the manuscript.

## Data sharing statement

The data used in this study are deidentified participant data and are not publicly available. Data may be requested from the Australian Institute of Health and Welfare (AIHW) by visiting https://www.aihw.gov.au/about-our-data/accessing-data-through-the-aihw/data-on-request. JAA, ACL, and RMH had full access to the data in the study and take responsibility for the integrity of the data and accuracy of data analysis.

## Declaration of interests

This work was supported by a Trevor B Kilvington Bequest (UTR 7.188), awarded by the University of Melbourne. SW, ST, and CC are on the Scientific Advisory Board for Diamedica Therapeutics. RMH (#1176922), ST (#2017897), and ACL (#1185467) receive salary support from the National Health and Medical Research Council. JAA and HGG were funded by Australian Government Research Training Scholarships (DOI: https://doi.org/10.82133/C42F-K220). HGG is supported by the National Health and Medical Research Council (#2022019) and the Norman Beischer Medical Research Foundation for unrelated research. ACL is supported by a Norman Beischer Medical Research Foundation clinical fellowship funding for unrelated research. JM is supported by the National Institute of Health Research for unrelated research. All other authors declare no competing interests.

## References

[1] Dimitriadis E., Rolnik D.L., Zhou W. (2023). Pre-eclampsia. Nat Rev Dis Primers.

[2] Duley L. (2009). The global impact of pre-eclampsia and eclampsia. Semin Perinatol.

[3] Abalos E., Duley L., Steyn D.W., Gialdini C. (2018). Antihypertensive drug therapy for mild to moderate hypertension during pregnancy. Cochrane Database Syst Rev.

[4] Tita A.T., Szychowski J.M., Boggess K. (2022). Treatment for mild chronic hypertension during pregnancy. N Engl J Med.

[5] Beech A., Mangos G. (2021). Management of hypertension in pregnancy. Aust Prescr.

[6] (2025). Pharmaceutical Benefits Scheme.

[7] (2025). Pharmaceutical Benefits Scheme.

[8] Duan L., Ng A., Chen W., Spencer H.T., Lee M.S. (2018). Beta-blocker subtypes and risk of low birth weight in newborns. J Clin Hypertens.

[9] Easterling T., Mundle S., Bracken H. (2019). Oral antihypertensive regimens (nifedipine retard, labetalol, and methyldopa) for management of severe hypertension in pregnancy: an open-label, randomised controlled trial. Lancet.

[10] Bone J.N., Sandhu A., Abalos E.D. (2022). Oral antihypertensives for nonsevere pregnancy hypertension: systematic review, network meta- and trial sequential analyses. Hypertension.

[11] Webster L.M., Myers J.E., Nelson-Piercy C. (2017). Labetalol versus nifedipine as antihypertensive treatment for chronic hypertension in pregnancy. Hypertension.

[12] Babbar K., Armo M., Bhanja R.L. (2015). A comparative study of efficacy of antihypertensive drugs and feto-maternal outcome in the treatment of pregnancy induced hypertension. Int J Reprod Contracept Obstet Gynecol.

[13] Ashworth D., Battersby C., Bick D. (2023). A treatment strategy with nifedipine versus labetalol for women with pregnancy hypertension: study protocol for a randomised controlled trial (Giant PANDA). Trials.

[14] Harper L.M., Kuo H.-C., Boggess K. (2025). Blood pressure control in pregnant patients with chronic hypertension and diabetes: should <130/80 be the target?. Am J Obstet Gynecol.

[15] Sanusi A.A., Leach J., Boggess K. (2024). Pregnancy outcomes of nifedipine compared with labetalol for oral treatment of mild chronic hypertension. Obstet Gynecol.

[16] Matthews A.A., Danaei G., Islam N., Kurth T. (2022). Target trial emulation: applying principles of randomised trials to observational studies. BMJ.

[17] Hernán M.A., Wang W., Leaf D.E. (2022). Target trial emulation: a framework for causal inference from observational data. JAMA.

[18] Hernán M.A. (2021). Methods of public health research - strengthening causal inference from observational data. N Engl J Med.

[19] Hernán M.A., Alonso A., Logan R. (2008). Observational studies analyzed like randomized experiments: an application to postmenopausal hormone therapy and coronary heart disease. Epidemiology.

[20] Hernán M.A., Robins J.M. (2016). Using big data to emulate a target trial when a randomized trial is not available. Am J Epidemiol.

[21] Admon A.J., Donnelly J.P., Casey J.D. (2019). Emulating a novel clinical trial using existing observational data. Predicting results of the PreVent Study. Ann Am Thorac Soc.

[22] Fu E.L. (2023). Target trial emulation to improve causal inference from observational data: what, why, and how?. J Am Soc Nephrol.

[23] Cashin A.G., Hansford H.J., Hernán M.A. (2025). Transparent reporting of observational studies emulating a target trial: the TARGET statement. BMJ.

[24] Society of Obstetric Medicine Australia and New Zealand (SOMANZ) (2024).

[25] Flood M.M., McDonald S.J., Pollock W.E., Davey M.-A. (2017). Data accuracy in the victorian perinatal data collection: results of a validation study of 2011 data. Health Inf Manag J.

[26] Australian Institute of Health and Welfare (2024). Pharmaceutical benefits scheme data collection. https://www.aihw.gov.au/about-our-data/our-data-collections/pharmaceutical-benefits-scheme.

[27] Chesnaye N.C., Stel V.S., Tripepi G. (2022). An introduction to inverse probability of treatment weighting in observational research. Clin Kidney J.

[28] Duffy J., Cairns A.E., Richards-Doran D. (2020). A core outcome set for pre-eclampsia research: an international consensus development study. BJOG.

[29] Gestational Hypertension and Preeclampsia: ACOG Practice Bulletin (2020). Number 222. Obstet Gynecol.

[30] National Institute for Health and Care Excellence (NICE) (2019). Hypertension in pregnancy: diagnosis and management. https://www.nice.org.uk/guidance/ng133.

[31] National Institute for Health and Care Excellence (NICE) (2019). Preterm labour and birth. https://www.nice.org.uk/guidance/ng25.

[32] Bergman J.E.H., Perraud A., Barišić I. (2024). Updated EUROCAT guidelines for classification of cases with congenital anomalies. Birth Defects Res.

[33] Hadlock F.P., Harrist R.B., Martinez-Poyer J. (1991). In utero analysis of fetal growth: a sonographic weight standard. Radiology.

[34] Etminan M., Brophy J.M., Collins G., Nazemipour M., Mansournia M.A. (2021). To adjust or not to adjust: the role of different covariates in cardiovascular observational studies. Am Heart J.

[35] Groenwold R.H.H., Palmer T.M., Tilling K. (2021). To adjust or not to adjust? When a "confounder" is only measured after exposure. Epidemiology.

[36] Rijnhart J.J.M., Lamp S.J., Valente M.J., MacKinnon D.P., Twisk J.W.R., Heymans M.W. (2021). Mediation analysis methods used in observational research: a scoping review and recommendations. BMC Med Res Methodol.

[37] Bartlett J.W., Hughes R.A. (2020). Bootstrap inference for multiple imputation under uncongeniality and misspecification. Stat Methods Med Res.

[38] von Hippel P.T., Bartlett J.W. (2021). Maximum likelihood multiple imputation: faster imputations and consistent standard errors without posterior draws. Stat Sci.

[39] Gordon H.G., Hiscock R.J., Shub A. (2025). Diabetes in pregnancy and school-age developmental outcomes for offspring: a statewide retrospective cohort study. Diabetes Care.

[40] Kennedy A.L., Hiscock R.J., Vollenhoven B.J. (2025). School-age outcomes among IVF and ICSI-conceived children: a causal inference analysis using linked population-wide data. BMC Med.

[41] Hiscock R.J., Atkinson J.A., Tong S. (2023). Educational outcomes for children at 7 to 9 years of age after birth at 39 vs 40 to 42 weeks' gestation. JAMA Netw Open.

[42] McCrum-Gardner E. (2008). Which is the correct statistical test to use?. Br J Oral Maxillofac Surg.

[43] Imbens G.W., Rubin D.B., Durlauf S.N., Blume L.E. (2010). Microeconometrics.

[44] Rosenbaum P.R., Rubin D.B. (1983). The central role of the propensity score in observational studies for causal effects. Biometrika.

[45] Austin P.C., Stuart E.A. (2015). Moving towards best practice when using inverse probability of treatment weighting (IPTW) using the propensity score to estimate causal treatment effects in observational studies. Stat Med.

[46] Austin P.C. (2009). Balance diagnostics for comparing the distribution of baseline covariates between treatment groups in propensity-score matched samples. Stat Med.

[47] StataCorp (2025).

[48] Linden A., Mathur M.B., VanderWeele T.J. (2020). Conducting sensitivity analysis for unmeasured confounding in observational studies using E-values: the e-value package. Stata J.

[49] VanderWeele T.J., Ding P. (2017). Sensitivity analysis in observational research: introducing the E-Value. Ann Intern Med.

[50] Countouris M., Mahmoud Z., Cohen J.B. (2025). Hypertension in pregnancy and postpartum: current standards and opportunities to improve care. Circulation.

[51] Dhali B., Bhattacharya S., Ganguly R.P., Bandyopadhyay S., Mondal M., Dutta M. (2012). A randomized trial of intravenous labetalol & oral nifedipine in severe pregnancy induced hypertension. Int J Reprod Contracept Obstet Gynecol.

[52] Shekhar S., Sharma C., Thakur S., Verma S. (2013). Oral nifedipine or intravenous labetalol for hypertensive emergency in pregnancy: a randomized controlled trial. Obstet Gynecol.

[53] Vermillion S.T., Scardo J.A., Newman R.B., Chauhan S.P. (1999). A randomized, double-blind trial of oral nifedipine and intravenous labetalol in hypertensive emergencies of pregnancy. Am J Obstet Gynecol.

[54] Shi D.D., Yang F.Z., Zhou L., Wang N. (2016). Oral nifedipine vs. intravenous labetalol for treatment of pregnancy-induced severe pre-eclampsia. J Clin Pharm Ther.

[55] Duro-Gómez J., Rodríguez-Marín A.B., Giménez de Azcárete M. (2017). A trial of oral nifedipine and oral labetalol in preeclampsia hypertensive emergency treatment. J Obstet Gynaecol.

[56] Zulfeen M., Tatapudi R., Sowjanya R. (2019). IV labetalol and oral nifedipine in acute control of severe hypertension in pregnancy-A randomized controlled trial. Eur J Obstet Gynecol Reprod Biol.

[57] Leonard S.A., Siadat S., Huybrechts K.F. (2025). Comparative effectiveness and safety of labetalol versus nifedipine for treatment of chronic hypertension during pregnancy. JACC Adv.

[58] Toto R.D., Tian M., Fakouhi K., Champion A., Bacher P. (2008). Effects of calcium channel blockers on proteinuria in patients with diabetic nephropathy. J Clin Hypertens.

[59] Smith A.C., Toto R., Bakris G.L. (1998). Differential effects of calcium channel blockers on size selectivity of proteinuria in diabetic glomerulopathy. Kidney Int.

[60] Konoshita T., Makino Y., Kimura T. (2013). A crossover comparison of urinary albumin excretion as a new surrogate marker for cardiovascular disease among 4 types of calcium channel blockers. Int J Cardiol.

[61] Dhaun N., Macintyre I.M., Melville V. (2009). Blood pressure-independent reduction in proteinuria and arterial stiffness after acute endothelin-a receptor antagonism in chronic kidney disease. Hypertension.

[62] Inoue S., Tomino Y. (2004). Effects of calcium antagonists in hypertensive patients with renal dysfunction: a prospective, randomized, parallel trial comparing benidipine and nifedipine. Nephrology.

[63] Kloke H.J., Wetzels J.F., Koene R.A., Huysmans F.T. (1998). Effects of low-dose nifedipine on urinary protein excretion rate in patients with renal disease. Nephrol Dial Transplant.

[64] Cativo E.H., Lopez P.D., Cativo D.P., Atlas S.A., Rosendorff C. (2021). The effect of calcium channel blockers on moderate or severe albuminuria in diabetic, hypertensive patients. Am J Med.

[65] Satoh M., Hirose T., Satoh H. (2022). Actual impact of angiotensin II receptor blocker or calcium channel blocker monotherapy on renal function in real-world patients. J Hypertens.

[66] Nishida Y., Takahashi Y., Tezuka K., Takeuchi S., Nakayama T., Asai S. (2017). Comparative effect of calcium channel blockers on glomerular function in hypertensive patients with diabetes mellitus. Drugs R&D.

[67] Boza B., Ersan F., Alpay V., Erenel H. (2026). The prognostic significance of proteinuria severity in pregnancy: a retrospective cohort study of maternal and neonatal outcomes. J Clin Med.

[68] Cravedi P., Remuzzi G. (2013). Pathophysiology of proteinuria and its value as an outcome measure in chronic kidney disease. Br J Clin Pharmacol.

[69] Nivethana K., Senthil P., Krupanidhi K. (2020). A comparative study of IV labetalol with oral nifedipine in severe preeclampsia. Int J Clin Obstet Gynecol.

[70] AIHW (Australian Institute of Health and Welfare) (2026). Australia's mothers and babies. https://www.aihw.gov.au/reports/mothers-babies/australias-mothers-babies/contents/about.

[71] Gunderson E.P., Greenberg M., Najem M. (2025). Severe maternal morbidity associated with chronic hypertension, preeclampsia, and gestational hypertension. JAMA Netw Open.

[72] Lowe S.A., Brown M.A., Dekker G.A. (2009). Guidelines for the management of hypertensive disorders of pregnancy 2008. Aust N Z J Obstet Gynaecol.

[73] Lowe S.A., Bowyer L., Lust K. (2015). SOMANZ guidelines for the management of hypertensive disorders of pregnancy 2014. Aust N Z J Obstet Gynaecol.

[74] Hernán M.A., Hernández-Díaz S., Robins J.M. (2004). A structural approach to selection bias. Epidemiology.

[75] Perejón D., Bardalet A., Gascó I., Siscart J., Serna M.C., Orós M. (2024). Hypertension subtypes and adverse maternal and perinatal outcomes - a retrospective population-based cohort study. BMC Pregnancy Childbirth.

[76] Eddings W., Marchenko Y. (2012). Diagnostics for multiple imputation in stata. Stata J.

[77] Rosenbaum P. (2017).

[78] Magee L.A., Abalos E., von Dadelszen P., Sibai B., Easterling T., Walkinshaw S. (2011). How to manage hypertension in pregnancy effectively. Br J Clin Pharmacol.

