# Supplementary material for: A comparison of the safety of oral labetalol versus nifedipine to manage hypertension in pregnancy in Australia: a target trial emulation
Source: eClinicalMedicine. 2026 Jun 5;96:104002. doi: 10.1016/j.eclinm.2026.104002 (PMC13266230; doi:10.1016/j.eclinm.2026.104002)
Supplement: Supplementary Material [file mmc1.docx]

**ONLINE SUPPLEMENTARY MATERIAL**

**A comparison of the safety of oral labetalol versus nifedipine to manage hypertensive disorders of pregnancy in Australia: A target trial emulation**

Jessica A ATKINSON BBiomed (Hons)^1,2^*, Anthea C LINDQUIST DPhil (Oxon)^1,2^*, Stephen TONG^1,2^, Richard J HISCOCK MD^1,2^, Anna FORSYTHE MEnv^1,2^, Hannah G GORDON MD^1,2^, Susan P WALKER MD^1,2^, Su Jen CHUA MD^1,2^, Catherine CLUVER PhD^3^, Jenny MYERS PhD^4^, Roxanne M HASTIE PhD^1,2^

1. Perinatal Epidemiology Group, Department of Obstetrics, Gynaecology, and Newborn Health, University of Melbourne, Melbourne, Victoria, AUSTRALIA.
2. Mercy Perinatal, Mercy Hospital for Women, Heidelberg, Victoria, AUSTRALIA.
3. SAMRC Extramural Preeclampsia Research Unit, Stellenbosch University, Cape Town, SOUTH AFRICA.
4. Maternal and Fetal Health Research Centre, Division of Developmental Biology and Medicine, University of Manchester, Manchester, UNITED KINGDOM.

* These authors contributed equally and are the co-first authors.

**SUPPLEMENTARY METHODS**

| **Page No.** | **Item** |
| --- | --- |
| 2 | Statistical analysis plan |
| 15 | Direct acyclic graphs for primary analyses |
| 16 | Details of imputation models |
| 18 | Details of primary analysis models |

**Supplementary Methods**

**Statistical Analysis Plan**

Version 2.3, September 2024

**BACKGROUND**

Hypertensive disorders of pregnancy affect up to 10% of pregnancies and are associated with a suite of adverse maternal and neonatal outcomes, including fetal growth restriction, preterm birth, and, in severe cases, maternal or neonatal mortality.^1,2^ There are currently no therapeutics available that can definitively treat hypertensive disorders of pregnancy. However, **antihypertensive agents** can be used to lower blood pressure and have been shown to reduce the risk of maternal complications. ^3^

In Australia and the United Kingdom, the two oral antihypertensive medications most commonly used in pregnancy are labetalol and nifedipine. ^4,5^ Both drugs are considered safe in pregnancy – however, it remains unclear which medication confers the lowest risk of adverse maternal, neonatal, and longer-term outcomes.^6^ Previous studies have been unable to establish a difference in the prevalence of outcomes related to use of nifedipine or labetalol, mainly due to the size of the study populations – they simply have not had the numbers needed to show clinically relevant differences associated with using one drug over the other.^7^

The Giant PANDA study is a pragmatic randomized trial in the early stages of recruitment in the United Kingdom. The aim of this trial is to compare nifedipine and labetalol to determine which is associated with optimal maternal and neonatal outcomes.^8^ These results will be critical in guiding future clinical practice but will not be available for several years. In the meantime, observational data may provide crucial insights.

Using state-wide linked data of over 1.2 million Victorian births and employing a formal approach to causal inference, we will determine whether labetalol or nifedipine is associated with improved maternal and perinatal outcomes. Using existing linked data, we will be able to provide critical evidence that may inform clinical practice, while the results of the Giant PANDA study are eagerly awaited.

**PROJECT OVERVIEW**

The study will be performed in two parts:

1. The demographic characteristics for women with a hypertensive disorder of pregnancy (i.e., chronic hypertension, gestational hypertension, or preeclampsia) and their infants will be described and compared based on pregnancy exposure to labetalol or nifedipine.
2. By applying a causal framework to the statistical analysis of our observational data, we will determine the *causal effect* of exposure to nifedipine for the management of hypertensive disorders of pregnancy, compared with exposure to labetalol, on maternal and perinatal adverse outcomes. This approach will be used to mimic the conduct of a target randomized controlled trial seeking to recruit pregnant women who require treatment with antihypertensives.
   1. **P**opulation – All women who gave birth in Victoria between 2009 – 2020 and whose pregnancies were affected by hypertensive disorders.
   2. **I**ntervention – Exposure to nifedipine from 11 weeks’ gestation.
   3. **C**ontrol – Exposure to labetalol from 11 weeks’ gestation.
   4. **O**utcome – Adverse maternal and neonatal outcomes (see *Outcome Definitions* below).
   5. **T**iming – From 11 weeks’ gestation until 28 days postpartum.

**DATA SOURCES**

1. Victorian Perinatal Data Collection (VPDC) and Births, Deaths, and Marriages Registry (BDM)

The VPDC contains comprehensive clinical data for every birth occurring in Victoria. This includes maternal, pregnancy, and infant characteristics and clinical outcomes. This dataset has been used to identify women who gave birth between **2009-2020** and their children. The VPDC has been validated with data from Births, Deaths and Marriages to ensure accuracy. From these datasets, we will ascertain key maternal and neonatal demographics and outcomes.

1. Pharmaceutical Benefits Scheme (PBS)

Many prescription medications are subsidized for Australian citizens under the Pharmaceutical Benefits Scheme, and each prescription medicine that is claimed under this scheme is contained within the PBS data collection. This dataset will be used to identify all women who were prescribed and dispensed labetalol or nifedipine during their pregnancies within the study period.

1. Victorian Admitted Episodes Dataset (VAED) and Victorian Emergency Minimum Dataset (VEMD)

The VAED collects data on all admitted patients from Victorian public and private hospitals. This includes information on presentations, diagnoses, and utilization of healthcare services. The VEMD collects detailed clinical information on all presentations within Victorian emergency departments, including the nature of illnesses/injuries, diagnoses, and transfers. The VEMD and VAED will be used to determine additional maternal and offspring demographics and health outcomes, which may not be included within the VPDC.

**PART ONE: DESCRIPTIVE ANALYSIS**

1. **Flow chart of participants**

Total number in cohort > number of linked records > number of hypertensive pregnancies exposed to labetalol or nifedipine.

**Exclusion criteria:** Use of both labetalol and nifedipine prior to 11 weeks’ gestation (unable to be randomized).

1. **Population characteristics to be described for:**
   1. Overall cohort
   2. Women using labetalol during pregnancy and their child(ren)
   3. Women using nifedipine during pregnancy and their child(ren)

| **Variable** | **Overall** | **Labetalol-Exposed** | **Nifedipine-Exposed** |
| --- | --- | --- | --- |
| **Maternal Baseline Characteristics** | | | |
| Maternal age (mean, SD)  *Missing (%)* |  |  |  |
| Maternal age >35 (%) |  |  |  |
| Marital status (%)  *Married/de facto*  *Single*  *Divorced*  *Widowed*  *Missing* |  |  |  |
| Parity (%)  *Nulliparous*  *Multiparous*  *Missing* |  |  |  |
| Hypertensive diagnosis (%)  *Chronic hypertension*  *Gestational hypertension*  *Preeclampsia* |  |  |  |
| Gestation at commencement of treatment (%)  *11^+0^ – 19^+6^ weeks*  *20^+0^ – 27^+6^ weeks*  *28^+0^ – 36^+6^ weeks* |  |  |  |
| Conception via assisted reproductive technology (%) |  |  |  |
| Body mass index (mean, SD)  *Missing (%)* |  |  |  |
| Body mass index ≥ 30 (%) |  |  |  |
| Maternal pre-existing comorbidities (%)  *Type 1 or 2 diabetes mellitus*  *Autoimmune disease*  *Renal disease*  *None* |  |  |  |
| Gestational diabetes mellitus (%)  *Yes*  *No*  *Missing* |  |  |  |
| SEIFA quintile (%)  *1 (most disadvantaged)*  *2*  *3*  *4*  *5 (least disadvantaged)*  *Missing* |  |  |  |
| **Neonatal Baseline Data** | | | |
| Sex (%)  *Male*  *Female*  *Missing* |  |  |  |
| Mode of birth (%)  *Elective caesarean section*  *Emergency caesarean section*  *Instrumental birth (forceps or vacuum)*  *Spontaneous vaginal birth*  *Missing* |  |  |  |
| Gestational age at birth (mean, SD)  *Missing* |  |  |  |
| Birthweight (mean, SD, centile)  *Missing (%)* |  |  |  |
| Plurality (%)  *Singleton*  *Twins or higher order births*  *Missing* |  |  |  |
| **Maternal Outcome Data** | | | |
| Maternal mortality (%) |  |  |  |
| Eclampsia (%) |  |  |  |
| HELLP Syndrome (%) |  |  |  |
| Stroke (%) |  |  |  |
| Cortical blindness (%) |  |  |  |
| Retinal detachment (%) |  |  |  |
| Pulmonary oedema (%) |  |  |  |
| Placental abruption (%) |  |  |  |
| Liver rupture (%) |  |  |  |
| Dialysis (%) |  |  |  |
| Extracorporeal membrane oxygenation (ECMO) (%) |  |  |  |
| Pregnancy prolongation index (PPI) (mean, SD) |  |  |  |
| Need for additional antihypertensives (%) |  |  |  |
| **Neonatal Outcome Data** | | | |
| Stillbirth (fetal death ≥ 20 weeks’ gestation) (%) |  |  |  |
| Neonatal death (death ≤ 28 days post-birth) (%) |  |  |  |
| Preterm birth (%)  *<37 weeks’ gestation*  *<34 weeks’ gestation*  *<32 weeks’ gestation*  *<28 weeks’ gestation* |  |  |  |
| Hadlock birthweight centile (mean, SD)  *≤10^th^ centile (%)*  *≤3^rd^ centile (%)* |  |  |  |
| Major congenital anomaly (EURO-CAT) (%) |  |  |  |
| Neonatal seizures (%) |  |  |  |
| Necrotizing enterocolitis (%) |  |  |  |
| Hypoxic ischemic encephalopathy (%) |  |  |  |
| Neonatal sepsis (%) |  |  |  |
| Intracranial hemorrhage (%) |  |  |  |
| Respiratory distress (%) |  |  |  |
| Neonatal asphyxia (%) |  |  |  |
| Neonatal cardiac arrest or heart failure (%) |  |  |  |
| Invasive resuscitation (%) |  |  |  |
| Non-invasive resuscitation (%) |  |  |  |

**PART TWO: CAUSAL ANALYSIS**

Combining the power of a large, linked state-wide dataset and causal inference methodology, we will examine whether there is a causal effect of exposure to nifedipine in pregnancy on adverse maternal or perinatal outcomes, when compared with exposure to labetalol.

*What is the causal effect of exposure to nifedipine in pregnancy on adverse maternal and neonatal outcomes, when compared with exposure to labetalol,* among women with hypertensive disorders of pregnancy?

**NULL HYPOTHESIS**

The incidence of maternal and neonatal adverse outcomes will be the same for women exposed to nifedipine during pregnancy, compared with those exposed to labetalol.

**ANTICIPATED SAMPLE SIZE**

Our sample will comprise all women with hypertensive disorders of pregnancy (chronic hypertension, gestational hypertension, or preeclampsia) who birthed in Victoria between 2009 – 2020, and were prescribed either labetalol or nifedipine between 11-36 weeks’ gestation. In a blinded review of our data, we have determined our sample size to be approximately 7,500. Of these, approximately 90% received labetalol, and the remaining 10% received nifedipine. Based on this, we are powered to detect an exact difference between groups of ≥3.0% for the maternal primary outcome and ≥5.6% for the neonatal primary outcome.

**CAUSAL ANALYSIS STRUCTURE**

**Analysis structure**

A target trial (TT) emulation framework will be used. Under this framework, the following are specified: (a) causal research question(s); (b) populations (target, source, and study population, set by eligibility criteria); (c) treatment strategy (exposure) and procedures to verify participants who received one of the treatments being studied; (d) follow-up period; (e) pre-specified primary and secondary outcomes, leading to specification of the causal estimand (contrast of interest) under the potential outcomes approach to causal inference; (f) determining that the causal estimand (the population quantity that we want to estimate) is identifiable within the observed data structure with corresponding choice of appropriate estimators. We consider that each estimator is defined by the steps (c) – (f) and propose to use several estimators in targeting the causal estimand (see Target Trial Emulation Table).

**Target Trial Emulation**

| **(A) Research Question** | ***What is the causal effect of exposure to nifedipine on adverse maternal and neonatal outcomes, compared with exposure to labetalol?*** | |
| --- | --- | --- |
|  | **Target Trial (Giant PANDA)** | **Emulation** |
| **(B) Populations** | All women with hypertensive disorders of pregnancy (chronic hypertension, gestational hypertension, or preeclampsia) requiring antihypertensive medication at gestational age 11^+0^-19^+6^; 20^+0^-27^+6^; and 28^+0^-36^+6^.  *Exclusions:* contraindication to labetalol or nifedipine; on both labetalol and nifedipine prior to randomization; unable to provide informed consent; unable to be randomized. | All women with hypertensive disorders of pregnancy (gestational hypertension, chronic hypertension, or preeclampsia) requiring antihypertensive treatment (dispensed labetalol or nifedipine) between 11^+0^ and 36^+6^ weeks’ gestation.  *Exclusions:* Taking both labetalol and nifedipine prior to 11 weeks’ gestation. |
| **(C) Treatment strategies** | Primary  Intention-to-treat (ITT): All women randomized to labetalol or nifedipine, regardless of discontinuation or crossover of treatment groups.    Sensitivity  Per-protocol: All women randomized to labetalol or nifedipine, who continued with assigned treatment until no longer indicated.  On-treatment: All women randomized to labetalol or nifedipine, who continued with assigned treatment until no longer indicated and who self-reported ≥90% adherence across all study contacts. | Primary  Intention-to-treat (ITT): All women who received ≥1 prescription and dispensation of labetalol or nifedipine, including those who received both medications during the study period (crossover will be determined by timing of dispensation) and those who discontinued treatment.  *Crossover defined as receiving both labetalol and nifedipine between 11-36 weeks’ gestation.*  Sensitivity  Per-protocol: All women who received ≥1 prescription and dispensation of labetalol or nifedipine during the study period, but did not receive both medications at any time. |
| **(C) Assignment procedures** | Randomized at diagnosis of hypertension requiring medical intervention (1:1 ratio of nifedipine/labetalol). | “Randomized” (via inverse probability weighting) at point of first dispensation of labetalol or nifedipine during the study period (≥11 weeks’ gestation and ≤36+6 weeks’ gestation). |
| **(D) Follow-up period** | From 11 weeks’ gestation until 28 days post-birth. | From 11 weeks’ gestation until 28 days post-birth. |
| **(E) Outcomes** | **Primary Maternal**  Severe hypertension (systolic blood pressure ≥160mmHg).  **Primary Neonatal**  Composite of stillbirth, neonatal death (until 28 days post-birth), and NICU admission requiring separation of infant and mother (until 28 days post-birth).  **Secondary Maternal**  *Note: Variables denoted with a * are reported with treatment effect and 95% CI, all others are exploratory only and described with summary statistics*.   - Mean antenatal systolic blood pressure* - New diagnosis of preeclampsia* - Severe maternal morbidity (fullPIERS consensus definition)* - Discontinuation of allocated antihypertensive* - Undesirable effects of allocated antihypertensive* - Maternal death* - Indicated birth (induction of PROM)* - Mean antenatal diastolic blood pressure - Proportion of days with antenatal systolic blood pressure ≥140mmHg - Proportion of days with antenatal diastolic blood pressure ≥90mmHg - Diagnosis of eclampsia - Diagnosis of HELLP syndrome - Placental abruption - Components of severe maternal morbidity - Maternal stroke - Prescription of additional antihypertensive drugs - Prescription of alternative antihypertensive drugs - Time from randomization to first discontinuation of allocated antihypertensive - Total number of antenatal hospital inpatient days - Treatment satisfaction - Beliefs about allocated antihypertensive drug - Adherence to allocated antihypertensive drug - Mode of onset of birth   **Secondary Neonatal**  *Note: Variables denoted with a * are reported with treatment effect and 95% CI, all others are exploratory only and described with summary statistics*.   - Major congenital anomaly (as defined by EURO-CAT)* - Birthweight centile* - Need for treatment for neonatal hypoglycemia* - Neonatal unit admission (separation of baby from mother)* - Mode of birth* - Gestational age at birth* - Fetal loss <24^+0^ weeks’ gestation - Fetal loss ≥24^+0^ weeks’ gestation (stillbirth) - Known early neonatal death (up to 7 days post-birth) - Known late neonatal death (7-28 days post-birth) - Principal recorded indication for neonatal unit admission - Length of stay in neonatal unit (and level of care) - Indication for mode of birth - Preterm birth (<37 completed weeks) - Preterm birth (<32 completed weeks) - Birthweight - Birthweight SGA (<10^th^ centile) - Umbilical arterial pH<7 at birth - Apgar score 5min post-delivery - Need for additional resuscitation at birth - Need for respiratory support - Type of respiratory support needed - Type of treatment for hypoglycemia - Lowest blood glucose measurement within 48 hours post-birth - Intracranial hemorrhage - Neonatal seizures - Necrotizing enterocolitis | **Primary Maternal**  Our primary outcome will be a composite of severe maternal morbidity determined by an expert panel, comprising:   - Maternal mortality (all cause) - Eclampsia - HELLP syndrome - Stroke - Cortical blindness - Retinal detachment - Pulmonary oedema - Placental abruption - Liver rupture - Need for dialysis - Need for extracorporeal membrane oxygenation (ECMO)   *Note: We do not have reliable ascertainment of ICU/HDU admission data. By instead including only severe morbidities which can reasonably be attributed to hypertensive disorders, we will provide a more accurate picture of the effect of disease-modifying drugs.*  **Primary Perinatal**  Our primary perinatal outcome will be a composite outcome determined by expert consensus. This will comprise.   - Stillbirth (fetal death ≥20 weeks’ gestation) - Neonatal death (death within 28 days of birth) - Severe neonatal morbidity, including:   - Bacterial sepsis   - Birthweight < 3^rd^ centile   - Cardiac arrest   - Hypoxic-ischemic encephalopathy (HIE)   - Intracranial hemorrhage   - Necrotizing enterocolitis   - Neonatal asphyxia   - Neonatal seizures   - Requirement for intubation or mechanical ventilation   - Requirement for resuscitation (mechanical or non-mechanical)   *Note: We do not have reliable ascertainment of NICU admission data. Many infants who are born following hypertensive pregnancies will be admitted to NICU without any other severe morbidities. By instead including only diagnosed severe morbidities, we will provide a more accurate picture of the effect of disease-modifying drugs.*  **Secondary Maternal**  *Note: Variables denoted with a * are reported with treatment effect and 95% CI, all others are exploratory only and described with summary statistics*.   - Pregnancy prolongation (time from first antihypertensive dispensation to birth)* - Need for additional antihypertensive(s)* - Individual components of the primary composite outcome - Disease progression (new diagnosis of preeclampsia after randomization)   **Secondary Perinatal**  *Note: Variables denoted with a * are reported with treatment effect and 95% CI, all others are exploratory only and described with summary statistics*. *** Indicates core secondary outcome.*   - Preterm birth (<37; <34; <32; and <28 weeks’ gestation)** - Major congenital anomaly (as defined by EURO-CAT)* - Neonatal hypoglycemia* - Respiratory distress - Neonatal hypothermia - Individual components of primary composite outcome - Birthweight <10^th^ centile |
| **(F) Causal contrasts of interest** | **Binary Outcomes**  Relative risk (RR) and risk difference (RD) of each outcome (point estimate RR 95% CI) | **Causal comparison:**  *What is the causal effect of exposure to nifedipine during pregnancy on adverse maternal and perinatal outcomes, compared with exposure to labetalol?*  **Estimand = Average treatment effect (ATE)**  Relative risk (RR) and risk difference (RD) of developing each adverse outcome (ATE RR 95% CI).  ***Estimator model*** *–*Doubly robust inverse probability weighting with regression adjustment. |

**Developing a causal model using directed acyclic graphs (DAGs) to both identify and summarise causal pathways.**

1. Potential confounders will be determined by the above-named authorship team (a quorum of specialists with expert knowledge). All potential confounders are pre-exposure or at exposure.
   1. Covariates to be included in the selection model:
      1. Type of hypertension (gestational hypertension, chronic hypertension, preeclampsia)*
      2. Maternal diabetes mellitus (yes/no)*
      3. Plurality (1/2+)*
      4. Pre-existing renal disease
      5. Pre-existing autoimmune disease
      6. Maternal smoking during pregnancy
      7. Maternal body mass index
      8. IVF/ART conception
      9. Maternal socioeconomic status (SEIFA quintile)
      10. Maternal age at delivery
      11. Mode of birth (neonatal outcomes only)

*Variables denoted with * are included in the Giant PANDA randomization algorithm.*

**Causal pathway and confounding** (excerpt from Causal Inference, What if. M Hernan & J Robins p85: <https://www.hsph.harvard.edu/miguel-hernan/causal-inference-book/>)

*“...the bias has the same structure: it is due to the presence of a cause (known covariate L or unknown covariate U) that is shared by the treatment A and the outcome Y, which results in an open backdoor path between A and Y. We refer to the bias caused by shared causes of treatment and outcome as confounding…”*

**Define populations (target, source, and study).**

- 1. The target trial population is all pregnant women, with access to obstetric services, who have diagnosed or suspected hypertensive disorders of pregnancy after 11 weeks’ gestation.
  2. The target trial source population is all women who gave birth in Victoria between 2009 – 2020.
  3. In the emulated target trial, our source population will include all hypertensive women (with gestational hypertension, chronic hypertension, or preeclampsia) who gave birth in Victoria between 2009 – 2020, who received a dispensation of labetalol or nifedipine between 11+0- and 36+6-weeks’ gestation.

**Define treatment strategy (exposure), assignment procedures, and follow-up period.**

1. The target trial would randomize at the point of diagnosis of a hypertensive disorder of pregnancy (chronic hypertension, gestational hypertension, or preeclampsia). Women would be randomized in a 1:1 ratio to either labetalol or nifedipine. Follow-up would be until 28 days postpartum, and reasons for drop out would include non-adherence to medication, failure to provide consent, unplanned crossover of participants, and random loss to follow up.
2. In the emulated trial, women will be ‘randomized’ at the point of first dispensation of either labetalol or nifedipine at or beyond 11 weeks’ gestation. We will exclude women who were taking both medications prior to 11 weeks’ gestation (unable to be randomized). We will exclude hypertensive women who received neither labetalol or nifedipine (contraindication or non-indication; this cohort will be described). We will also exclude women without hypertension who received labetalol or nifedipine (this cohort will be described).

**Outcome definitions – primary and secondary outcomes.**

1. In the target trial, the **primary maternal outcome** is severe hypertension (systolic blood pressure ≥160mmHg). We do not have reliable access to blood pressure measurements; however, our much larger sample size allows us to investigate downstream effects of severe hypertension (severe morbidity). Therefore, the primary maternal outcome will be a composite comprising:
   1. Maternal mortality (all cause) (yes/no)
   2. Eclampsia (yes/no)
   3. HELLP syndrome (yes/no)
   4. Stroke (yes/no)
   5. Cortical blindness (yes/no)
   6. Retinal detachment (yes/no)
   7. Pulmonary oedema (yes/no)
   8. Placental abruption (yes/no)
   9. Liver rupture (yes/no)
   10. Need for dialysis (yes/no)
   11. Need for extracorporeal membrane oxygenation (ECMO) (yes/no)
2. In the target trial, the **primary perinatal outcome** is a composite of stillbirth (≥20 weeks’ gestation), neonatal death (≤28 days post-birth), and neonatal unit admission requiring separation of the infant and mother. We will maintain this outcome by reporting a primary perinatal outcome comprising:
   1. Stillbirth ≥ 20 weeks’ gestation (yes/no)
   2. Neonatal death ≤ 28 days post-birth (yes/no)
   3. Severe neonatal morbidity, comprising:
      1. Neonatal seizures (yes/no)
      2. Necrotizing enterocolitis (yes/no)
      3. Hypoxic-ischemic encephalopathy (yes/no)
      4. Birthweight <3^rd^ centile (yes/no)
      5. Bacterial sepsis (yes/no)
      6. Intracranial hemorrhage (yes/no)
      7. Neonatal asphyxia
      8. Cardiac arrest (yes/no)
      9. Requirement for mechanical or non-mechanical resuscitation (yes/no)
      10. Requirement for intubation or mechanical ventilation (yes/no)
3. In the target trial, there were several **secondary maternal outcomes** (see *Target Trial Emulation* Table Sfor full list). As these secondary outcomes are largely accounted for in our primary composite outcome, we will report the following secondary outcomes with treatment effect and 95% confidence intervals (CI):
   1. Need for additional antihypertensives (yes/no)
   2. Progression from gestational hypertension to preeclampsia (yes/no)

Additionally, the following outcomes will also be reported in an exploratory capacity (i.e., with summary statistics only):

1. Individual components of the primary outcome (all reported as yes/no)
2. Hospital admission prior to 37 weeks’ gestation (yes/no)
3. Total number of dose(s) of allocated antihypertensive drug (mean, SD)
4. In the target trial, there were several **secondary perinatal outcomes** (see *Target Trial Emulation* Table Sfor full list). In our emulation, we will report the following secondary outcomes with treatment effect and 95% confidence intervals (CI):
   1. Preterm birth <37; <34; <32; and <28 weeks’ gestation (yes/no)
   2. Birthweight ≤3^rd^ centile (yes/no)
   3. Neonatal hypoglycemia (yes/no)
   4. Major congenital anomaly as defined by EURO-CAT (yes/no)

Additionally, the following outcomes will also be reported in an exploratory capacity (i.e., with summary statistics only):

1. Individual components of the primary composite outcome (all yes/no)
2. Birthweight <10^th^ centile (yes/no)
3. Mode of birth (spontaneous vaginal, operative vaginal, elective caesarean section, emergency caesarean section)

**Causal contrasts of interest**

1. **Estimand**

**Maternal outcomes** – relative risk (RR) and risk difference (RD) of primary composite outcome and pre-specified (*) secondary outcomes (ATE RR, 95% CI).

**Neonatal outcomes** – relative risk (RR) and risk difference (RD) of primary composite outcome and pre-specified (*) secondary outcomes (ATE RR, 95% CI).

In the Giant PANDA RCT, the primary neonatal binary outcome is estimated using a type of logistic regression (log-binomial) model. We will use a logistic regression model with robust standard errors (to adjust for maternal clustering). This model provides predictive probabilities that are then used to calculate both the between exposure difference (RD) and relative risk (RR) for all binary and highlighted (*) secondary outcomes.

In our emulated target trial, the estimand is the average treatment effect (ATE) between exposures, presented using both RD and RR metrics. Within the potential outcomes framework, a causal interpretation can be made under the assumptions of counterfactual consistency, ignorability (conditional exchangeability), and positivity. Counterfactual consistency means that the definition of exposure is consistent for all individuals. Ignorability states that treatment assignment can be considered exchangeable (i.e. as if the assignment was randomized) after controlling for, or conditioning on, a set of covariates. This implies that there are no important unmeasured confounders, an untestable assertion. The positivity assumption means that, for all observations, the conditional probability of being exposed and of not being exposed (receiving treatment or not receiving treatment) are both greater than zero.

1. **Estimators**

In the emulated target trial, all estimators are targeting the ATE estimand detailed above. Each estimator is specific to the complete process used from the input (observational dataset) to model-based calculation of the ATE. These include: the methods and detailed modelling used in handling missing data; the analysis model used for outcome estimation; compatibility between the imputation and outcome models; and methods used to estimate variance when combining results across imputations and/or bootstrap samples. In the setting of complete data (following multiple imputation) both parametric doubly robust (DR) or non-parametric DR (e.g., machine learning data adaptive) methods, that combine selection and outcome regression models, can provide minimally biased ATE estimates if one of these regression models is correct. Valid 95% confidence limits require that uncertainties in both imputation and outcome models are accounted for (*vide infra*).

**Handling of missing data**

Detailed description of the patterns of missing data will be provided. This will include the proportion of missingness for each analysis model covariate and those used in the construction of the minimization criteria. We will not impute missing exposure or outcome data. Mothers with missing exposure data (i.e., no record of prescription of either labetalol or nifedipine) will be excluded from analysis. The magnitude and characteristics of these mothers will be summarized in a supplementary table.

**Handling of missing data using multiple imputation**

Multiple imputation will be performed using fully conditional specification (FCS) and the imputed datasets will then be used for the outcome analysis. Covariates that will be included in the imputation model are: all outcome covariates, exposure, the Giant PANDA minimization variables (excluding obstetric unit identifier and self-reported Black ethnicity), all covariates included in the selection and outcome models irrespective of missingness, and exposure and auxiliary covariates associated with the outcome.

It is important that the models used in the imputation phase are compatible with those used for the ATE estimation. This requirement means that choice of imputation model requires knowledge of the analysis model and the covariates used in both selection and/or outcome models. This ensures that there is compatibility between the two. This is an open area of active research, with methods chosen based upon current recommendations in the literature. Given our dataset is likely to have >50% missingness for some variables, it may not be possible to include these covariates in the model. These issues will be formally noted, along with the details of the explicit modelling choices made, and appropriate sensitivity analyses will be undertaken. When an interaction is possible between exposure and outcome model covariates, these will be included in the imputation model.

Standard imputation diagnostics will then be performed to assess the quality of the imputed datasets.

**Parametric and non-parametric causal models:** Potential estimators using multiple imputation (MI) datasets are (A) selection model, using inverse probability weighting (IPW); (B) outcome model, using regression adjustment (RA); (C) doubly robust augmented inverse probability weighting regression adjustment model (IPWRA); and (D) doubly robust targeted maximum likelihood estimation (TMLE) using machine learning algorithms.

**(A) Selection model using inverse probability weights (IPW)**

We will model exposure assignment using inverse probability weighted propensity scores, derived from the logistic exposure model, and including the minimization variables. Adequacy of balance achieved by propensity score weighting will be assessed using: (i) standardized mean difference for model covariates and variance estimates between exposure groups; and (ii) propensity scores overlap, to assess positivity assumption. A finding of lack of positivity will lead to measures such as: addition of interaction terms; different exposure models; use of alternate weighting structures (overlap or stabilized); and lastly, propensity score-based trimming, with the aim of achieving overlap in the propensity score distributions.

**(B) Outcome model using regression adjustment (RA)**

We will use a regression adjustment outcome model, incorporating appropriate variables as determined from directed acyclic graph (DAG) analysis.

**(C) Augmented inverse probability weighted regression adjustment model (IPWRA)**

This is one of the doubly robust methods which combines the IPW selection model with the RA outcome model. This provides two opportunities for correct model specification and may provide valid estimates if only one of the submodels is mis-specified.

**(D) Doubly robust targeted maximum likelihood estimation (TMLE), using machine learning algorithms**

TMLE model estimation uses the SuperLearner ensemble learning method. ^9,10^ This combines prediction to choose which algorithm within the library to use, based upon a weighting approach where weights are proportional to prediction performance for each algorithm assessed using cross-validation. TMLE uses data adaptive methods outlined in four steps^11^ and incorporates the pre-specification of a flexible suite of algorithms:

(i) Estimate model for expected outcome conditional on exposure and confounder set, and then use this model to predict the outcome for all records under exposure and non-exposure.

(ii) Estimate selection model for probability of exposure conditional on the confounder set.

(iii) Incorporate the PS information from step (ii) to improve the initial outcome prediction, so that they solve the parameter’s efficient influence curve – the smallest variance in the target parameter. The influence function is a function that describes the estimated behavior when the empirical distribution of the data is slightly perturbed.

(iv) Plus the updated prediction in the g-formulation (RA model) to estimate the ATE.

**Combining individual analysis model ATE into the population ATE estimator.**

Probability weighting induces within subject correlation in the outcome in the weighted sample resulting in biased (on the low side) standard errors (SE’s). While a robust SE setting in the analysis model does account for this lack of independence, it does not account for the fact that the propensity score is estimated, rather than known.

Alternatives include:

(i) Bootstrapping, where the propensity score is estimated within each bootstrap sample, and thus the bootstrap estimate of the standard error incorporates sampling variability in the estimated propensity score (BootImpute). ^12^

(ii) BootImpute von Hippel^13^, where the ATE variance estimate is calculated from an ANOVA model, and requires a smaller number of bootstrap samples and imputations.

We will integrate IPW with multiple imputation to calculate the pooled ATE. This will be performed using the BootMI von Hippel method. ^13^

In brief, we obtain B (B=1000) bootstrapped samples from the observational data; for each of the B bootstrapped samples, impute M datasets (M=2); the ATE point estimate is calculated as mean over all B times M complete datasets; a one-way ANOVA model is used to estimate the two variance components (within and between bootstrap samples), which are then used to estimate the standard error and construct associated 95% confidence intervals, incorporating Satterthwaite’s degrees of freedom correlation.

Consideration will be given to using Boot MI percentile, if computationally possible as a sensitivity analysis. In this procedure the first step is to obtain B (B=1000) bootstrapped samples from the observational data; for each of the B bootstrapped samples, impute M datasets (M=20 to 50); a point estimate is then calculated, and a 95% percentile-based confidence interval for the population parameter is generated by taking 2.5% and 97.5% empirical percentiles based upon the mean ATE of the M imputed datasets for each bootstrapped sample. ^12^

**PART THREE: SUBGROUP ANALYSES**

Subgroup analyses will be undertaken by:

- Type of hypertensive disorder (chronic hypertension, gestational hypertension, and preeclampsia).
- Per-protocol and complete case analyses.
- Timing of randomization (11+0 – 19+6; 20+0 – 27+6; 28+0 – 36+0).
- Timing of birth (≤34 weeks’ gestation; >34 weeks’ gestation).

**SOFTWARE**

Analysis will be performed using Stata v18 (StataCorp. 2023. *Stata Statistical Software: Release 18*. College Station, TX: StataCorp LLC). Causal inference will be performed using *teffects* commands (treatment-effects estimation for observational data, see TE-teffects) run within Stata’s multiple imputation suite and TMLE using the *etmle* module^9^ or *TMLE* package in R.

**REFERENCES**

1. Khedagi AM, Bello NA. Hypertensive Disorders of Pregnancy. *Cardiol Clin*. 2021;39:77-90. doi: 10.1016/j.ccl.2020.09.005

2. Duley L. The Global Impact of Pre-eclampsia and Eclampsia. *Seminars in Perinatology*. 2009;33:130-137. doi: https://doi.org/10.1053/j.semperi.2009.02.010

3. Brown CM, Garovic VD. Drug treatment of hypertension in pregnancy. *Drugs*. 2014;74:283-296. doi: 10.1007/s40265-014-0187-7

4. Beech A, Mangos G. Management of hypertension in pregnancy. *Aust Prescr*. 2021;44:148-152. doi: 10.18773/austprescr.2021.039

5. National Institute for Health and Care Excellence: Guidelines. In: *Hypertension in pregnancy: diagnosis and management*. London: National Institute for Health and Care Excellence (NICE) Copyright © NICE 2019.; 2019.

6. Abalos E, Duley L, Steyn DW, Gialdini C. Antihypertensive drug therapy for mild to moderate hypertension during pregnancy. *Cochrane Database Syst Rev*. 2018;10:Cd002252. doi: 10.1002/14651858.CD002252.pub4

7. Bone JN, Sandhu A, Abalos ED, Khalil A, Singer J, Prasad S, Omar S, Vidler M, von Dadelszen P, Magee LA. Oral Antihypertensives for Nonsevere Pregnancy Hypertension: Systematic Review, Network Meta- and Trial Sequential Analyses. *Hypertension*. 2022;79:614-628. doi: 10.1161/hypertensionaha.121.18415

8. Ashworth D, Battersby C, Bick D, Green M, Hardy P, Leighton L, Magee LA, Maher A, McManus RJ, Moakes C, et al. A treatment strategy with nifedipine versus labetalol for women with pregnancy hypertension: study protocol for a randomized controlled trial (Giant PANDA). *Trials*. 2023;24:584. doi: 10.1186/s13063-023-07582-9

9. Luque-Fernandez MA. *ELTMLE: Stata module to provide Ensemble Learning Targeted Maximum Likelihood Estimation;* Boston: Boston College Department of Economics; 2017.

10. Luque-Fernandez MA, Schomaker M, Rachet B, Schnitzer ME. Targeted maximum likelihood estimation for a binary treatment: A tutorial. *Stat Med*. 2018;37:2530-2546. doi: 10.1002/sim.7628

11. Dashti SG, Lee KJ, Simpson JA, White IR, Carlin JB, Moreno-Betancur M. Handling missing data when estimating causal effects with targeted maximum likelihood estimation. *American Journal of Epidemiology*. 2024. doi: 10.1093/aje/kwae012

12. Bartlett JW, Hughes RA. Bootstrap inference for multiple imputation under uncongeniality and misspecification. *Stat Methods Med Res*. 2020;29:3533-3546. doi: 10.1177/0962280220932189

13. von Hippel PT, Bartlett JW. Maximum Likelihood Multiple Imputation: Faster Imputations and Consistent Standard Errors Without Posterior Draws. *Statistical Science*. 2021;36:400-420, 421.

**Direct Acyclic Graphs (DAGs) for primary analyses**


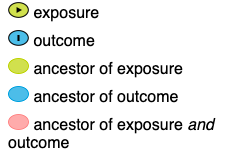

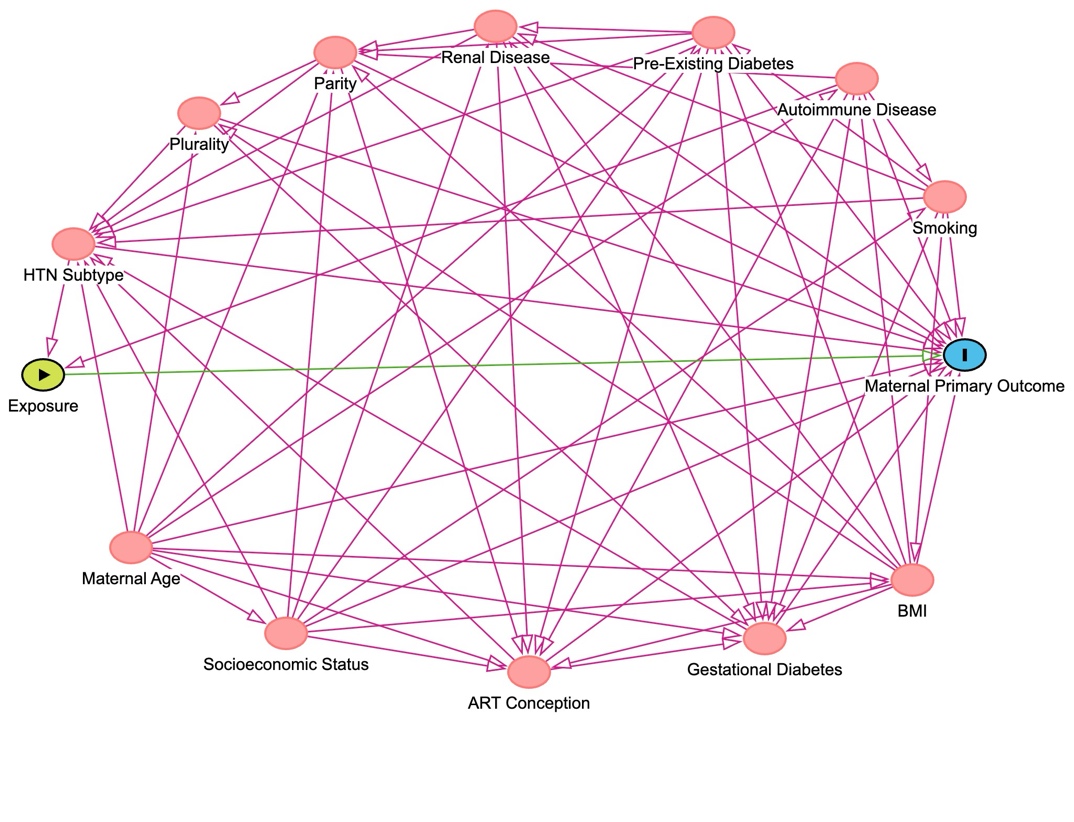


1. Direct Acyclic Graph (DAG) for maternal primary composite outcome


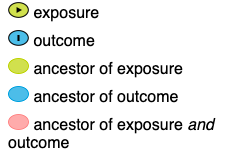

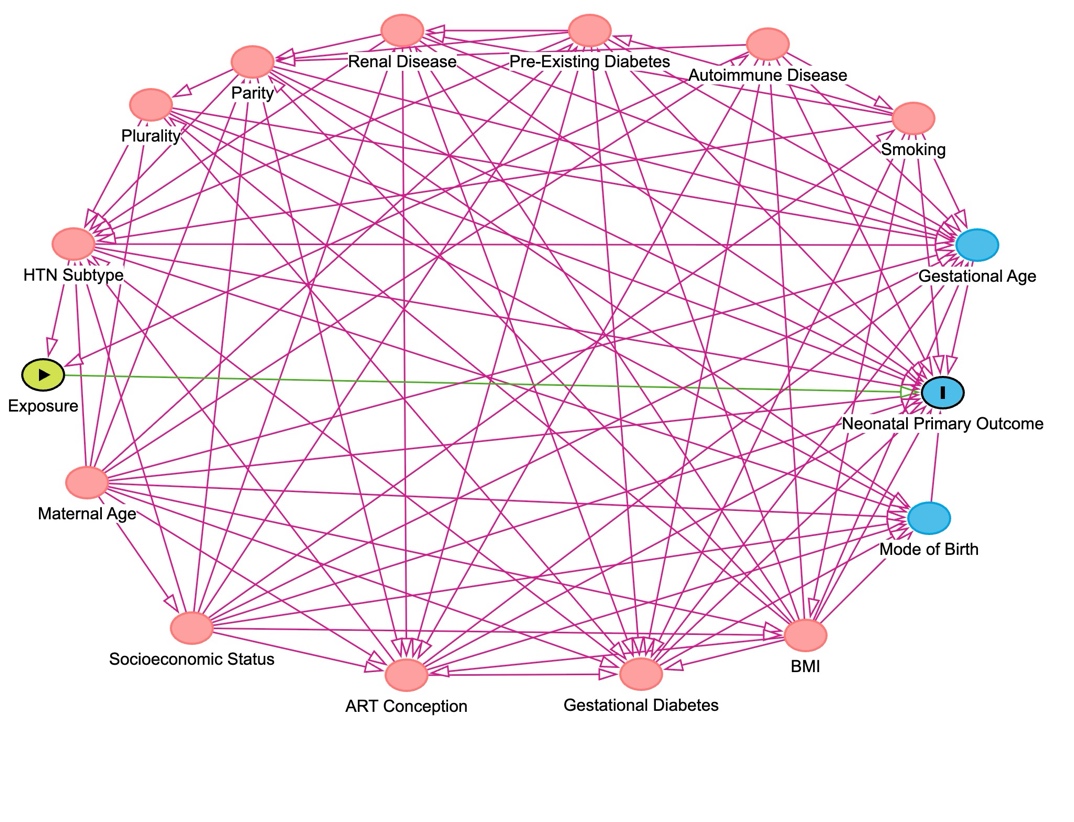


1. Direct Acyclic Graph (DAG) for neonatal primary composite outcome

**Details of imputation models**

|  | Observations per m | | | |
| --- | --- | --- | --- | --- |
| Variable | Complete | Incomplete | Imputed | Total |
| BMI | 7174 | 371 | 371 | 7545 |
| Smoking | 7471 | 74 | 74 | 7545 |
| SEIFA | 7542 | 3 | 3 | 7545 |
| IVF/ART Conception | 7521 | 24 | 24 | 7545 |
| Birthweight | 7532 | 13 | 13 | 7545 |

Observed raw data

| Variable | N | Mean | SD | Min | Max |
| --- | --- | --- | --- | --- | --- |
| BMI | 7174 | 31.71 | 7.50 | 10.01 | 59.88 |
| Smoking | 7471 | 0.16 | 0.37 | 0 | 1 |
| SEIFA | 7542 | 3.13 | 1.31 | 1 | 5 |
| IVF/ART Conception | 7521 | 0.09 | 0.28 | 0 | 1 |
| Birthweight | 7532 | 2926.36 | 734.23 | 160 | 5860 |

Imputed data bootstrap 1, m=1

| Variable | N | Mean | SD | Min | Max |
| --- | --- | --- | --- | --- | --- |
| BMI | 7545 | 31.69 | 7.49 | 10.01 | 59.88 |
| Smoking | 7545 | 0.16 | 0.37 | 0 | 1 |
| SEIFA | 7545 | 3.13 | 1.31 | 1 | 5 |
| IVF/ART Conception | 7545 | 0.09 | 0.28 | 0 | 1 |
| Birthweight | 7545 | 2922.03 | 741.39 | 160 | 5860 |

Imputed data bootstrap 2, m=2

| Variable | N | Mean | SD | Min | Max |
| --- | --- | --- | --- | --- | --- |
| BMI | 7545 | 31.69 | 7.51 | 10.01 | 59.88 |
| Smoking | 7545 | 0.17 | 0.37 | 0 | 1 |
| SEIFA | 7545 | 3.13 | 1.31 | 1 | 5 |
| IVF/ART Conception | 7545 | 0.09 | 0.28 | 0 | 1 |
| Birthweight | 7545 | 2922.11 | 741.21 | 160 | 5860 |

Proportions for SEIFA (m=1)

| **SEIFA Quintile** | **Observed** | **Imputed** | **Completed** |
| --- | --- | --- | --- |
| 1 | 0.154 | 0.333 | 0.154 |
| 2 | 0.163 | 0.333 | 0.163 |
| 3 | 0.266 | 0.000 | 0.265 |
| 4 | 0.237 | 0.000 | 0.237 |
| 5 | 0.180 | 0.333 | 0.180 |

Proportions for smoking status (m=1)

| **Smoking Status** | **Observed** | **Imputed** | **Completed** |
| --- | --- | --- | --- |
| Yes | 0.165 | 0.122 | 0.164 |
| No | 0.835 | 0.878 | 0.836 |

Proportions for IVF/ART conception (m=1)

| **IVF/ART Conception** | **Observed** | **Imputed** | **Completed** |
| --- | --- | --- | --- |
| Yes | 0.087 | 0.208 | 0.088 |
| No | 0.913 | 0.792 | 0.912 |

Proportions for BMI (m=1)


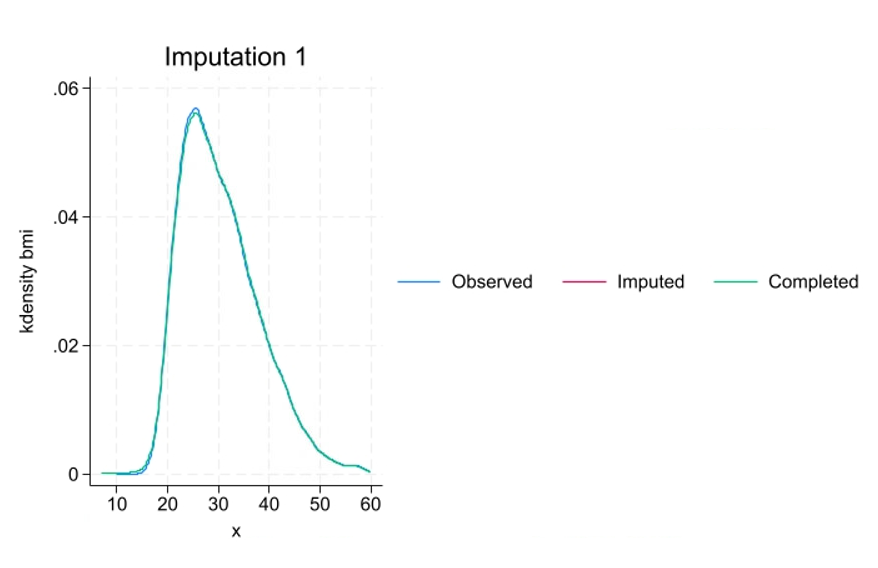


Proportions for birthweight (m=1)


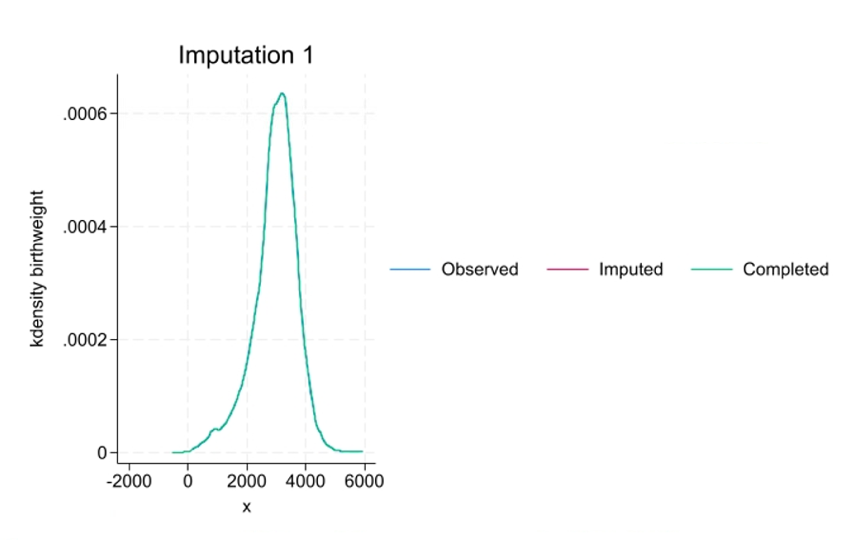


**Details of primary analysis models**

1) Maternal primary analysis model: Propensity score covariate balance assessment, pooled across all imputations and bootstraps

| **Variable** | **Standardised Differences** | |
| --- | --- | --- |
|  | **Raw** | **Weighted** |
| Hypertensive subtype |  |  |
| *Gestational hypertension (ref)* | – | – |
| *Chronic hypertension* | 0.0536 | 0.0046 |
| *Preeclampsia* | 0.1222 | 0.0058 |
| Maternal comorbidities |  |  |
| *No (Ref)* | – | – |
| *Yes* | 0.1745 | 0.0075 |
| Parity |  |  |
| *Multiparous (Ref)* | – | – |
| *Nulliparous* | 0.1103 | 0.0370 |
| Smoking in pregnancy |  |  |
| *No (Ref)* | – | – |
| *Yes* | 0.0062 | 0.0124 |
| Plurality |  |  |
| *Singleton (Ref)* | – | – |
| *Twins or higher order* | 0.0666 | -0.0051 |
| ART/IVF Conception |  |  |
| *No (Ref)* | – | – |
| *Yes* | 0.1759 | -0.0057 |
| SEIFA Quintile |  |  |
| *1 (Ref)* | – | – |
| *2* | -0.0095 | -0.0123 |
| *3* | -0.0933 | -0.0143 |
| *4* | 0.0299 | -0.0007 |
| *5* | 0.1331 | 0.0153 |
| Maternal age | 0.1623 | 0.0012 |
| Maternal body mass index | -0.0366 | 0.0193 |

Test of overidentifying restrictions: chi2(11) = 17.67

Prob > chi2 = 0.0894

Covariates are balanced.

1. Neonatal primary analysis model: Propensity score covariate balance assessment, pooled across all imputations and bootstraps

| **Variable** | **Standardised Differences** | |
| --- | --- | --- |
|  | **Raw** | **Weighted** |
| Hypertensive subtype |  |  |
| *Gestational hypertension (ref)* | – | – |
| *Chronic hypertension* | 0.0459 | 0.0053 |
| *Preeclampsia* | 0.1419 | 0.0003 |
| Maternal comorbidities |  |  |
| *No (Ref)* | – | – |
| *Yes* | 0.1675 | 0.0083 |
| Parity |  |  |
| *Multiparous (Ref)* | – | – |
| *Nulliparous* | 0.0940 | 0.0413 |
| Smoking in pregnancy |  |  |
| *No (Ref)* | – | – |
| *Yes* | 0.0081 | 0.0132 |
| Plurality |  |  |
| *Singleton (Ref)* | – | – |
| *Twins or higher order* | 0.0988 | -0.0101 |
| ART/IVF Conception |  |  |
| *No (Ref)* | – | – |
| *Yes* | 0.1889 | -0.0100 |
| SEIFA Quintile |  |  |
| *1 (Ref)* | – | – |
| *2* | -0.0174 | -0.0108 |
| *3* | -0.0936 | -0.0136 |
| *4* | 0.0289 | -0.0027 |
| *5* | 0.1465 | 0.0132 |
| Maternal age | 0.1702 | -0.0006 |
| Maternal body mass index | -0.0260 | 0.0155 |

Test of overidentifying restrictions: chi2(10) = 15.23

Prob > chi2 = 0.1236

Covariates are balanced.

1. Propensity score overlap plots for primary maternal and neonatal analyses, across all imputations (M=0, M=1, M=2)

| **Maternal Analyses** | | |
| --- | --- | --- |
| 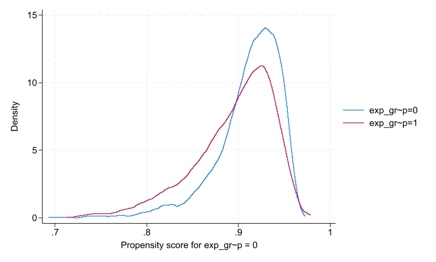  M=0 | 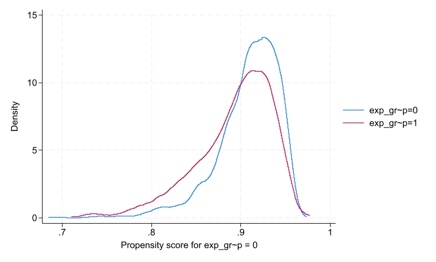  M=1 | 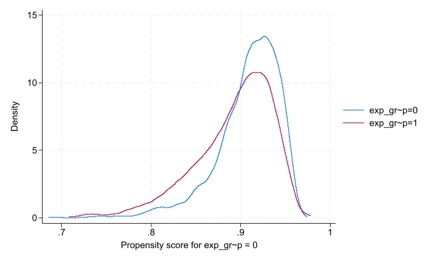  M=2 |
| **Neonatal Analyses** | | |
| 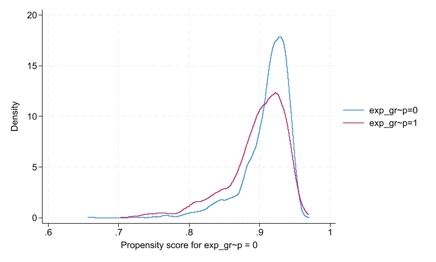  M=0 | 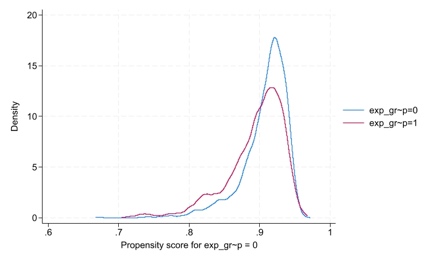  M=1 | 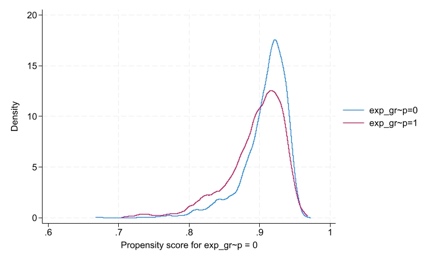  M=2 |
