# Supplementary material for: A comparison of the safety of oral labetalol versus nifedipine to manage hypertension in pregnancy in Australia: a target trial emulation
Source: eClinicalMedicine. 2026 Jun 5;96:104002. doi: 10.1016/j.eclinm.2026.104002 (PMC13266230; doi:10.1016/j.eclinm.2026.104002)
Supplement: Supplementary Tables [file mmc2.docx]

**ONLINE SUPPLEMENTARY MATERIAL**

**A comparison of the safety of oral labetalol versus nifedipine to manage hypertensive disorders of pregnancy in Australia: A target trial emulation**

Jessica A ATKINSON BBiomed (Hons)^1,2^*, Anthea C LINDQUIST DPhil (Oxon)^1,2^*, Stephen TONG^1,2^, Richard J HISCOCK MD^1,2^, Anna FORSYTHE MEnv^1,2^, Hannah G GORDON MD^1,2^, Susan P WALKER MD^1,2^, Su Jen CHUA MD^1,2^, Catherine CLUVER PhD^3^, Jenny MYERS PhD^4^, Roxanne M HASTIE PhD^1,2^

1. Perinatal Epidemiology Group, Department of Obstetrics, Gynaecology, and Newborn Health, University of Melbourne, Melbourne, Victoria, AUSTRALIA.
2. Mercy Perinatal, Mercy Hospital for Women, Heidelberg, Victoria, AUSTRALIA.
3. SAMRC Extramural Preeclampsia Research Unit, Stellenbosch University, Cape Town, SOUTH AFRICA.
4. Maternal and Fetal Health Research Centre, Division of Developmental Biology and Medicine, University of Manchester, Manchester, UNITED KINGDOM.

* These authors contributed equally and are the co-first authors.

**SUPPLEMENTARY TABLES**

| **Page No.** | **Item** |
| --- | --- |
| 2 | **Table S1.** Complete case sensitivity analysis |
| 3 | **Table S2.** Per-protocol sensitivity analysis |
| 4 | **Table S3.** Sensitivity analysis, stratified by timing of randomisation |
| 5 | **Table S4.** Sensitivity analysis, stratified by timing of birth (neonatal outcomes) |
| 6 | **Table S5.** Sensitivity analysis, adjusting for year of delivery |
| 7 | **Table S6.** Sensitivity analysis, excluding women with asthma |
| 8 | **Table S7.** Sensitivity analysis, restricting to first pregnancy episode per woman |
| 9 | **Table S8.** Sensitivity analysis using propensity score matching |
| 10 | **Table S9.** List of definitions for primary and secondary outcomes |
| 12 | **Table S10.** E-value for point estimates and 95% confidence interval closest to the null value for the risk ratios (RR) and risk differences (RD) of statistically significant primary and secondary outcomes |

**Table S1. Complete case sensitivity analysis**

|  | **Observed proportions** | | **Unadjusted effect estimates** | | **Adjusted effect estimates^a^** | |
| --- | --- | --- | --- | --- | --- | --- |
|  | **Labetalol (%)** | **Nifedipine (%)** | **Risk difference (95% CI) (%)** | **Risk ratio (95% CI)** | **Risk difference (95% CI) (%)** | **Risk ratio (95% CI)** |
| **Composite Maternal Outcome** | | | | | | |
| All hypertensive disorders | 413 (6.5) | 57 (9.3) | 2.85 (0.46, 5.23) | 1.44 (1.11, 1.87) | 2.09 (-0.20, 4.39) | 1.32 (1.01, 1.74) |
| Preeclampsia^b^ | 255 (11.7) | 41 (16.9) | 5.15 (0.25, 10.0) | 1.44 (1.06, 1.95) | 4.51 (-0.43, 9.46) | 1.39 (1.01, 1.90) |
| Gestational or chronic hypertension^b^ | 158 (3.8) | 16 (4.4) | 0.58 (-1.58, 2.75) | 1.16 (0.70, 1.91) | 0.46 (-1.68, 2.61) | 1.12 (0.67, 1.87) |
| **Composite Neonatal Outcome** | | | | | | |
| All hypertensive disorders | 2743 (42.4) | 283 (45.1) | 2.75 (-1.33, 6.82) | 1.06 (0.97, 1.17) | -0.94 (-4.98, 3.10) | 0.99 (0.89, 1.08) |
| Preeclampsia^b^ | 1153 (51.8) | 137 (53.1) | 1.26 (-5.18, 7.69) | 1.02 (0.91, 1.16) | -2.01 (-8.57, 4.56) | 0.96 (0.84, 1.10) |
| Gestational or chronic hypertension^b^ | 1590 (37.4) | 146 (39.6) | 2.13 (-3.07, 7.33) | 1.06 (0.93, 1.21) | -0.77 (-5.79, 4.24) | 0.98 (0.86, 1.12) |

*^a^ All models adjusted for the following: maternal age at delivery; maternal body mass index; marital status; conception via artificial reproductive technology; maternal diabetes mellitus (gestational or pre-existing); maternal pre-existing renal disease; smoking during pregnancy; socioeconomic status; plurality; and parity. Neonatal models additionally adjusted for gestational age at birth and mode of delivery. Complete-case analysis excludes all participants with missing data.*

***Maternal composite outcome:*** *maternal mortality (all-cause); eclampsia; HELLP syndrome; stroke; cortical blindness; retinal detachment; pulmonary oedema; placental abruption; liver rupture; renal failure requiring dialysis; heart or respiratory failure requiring extracorporeal membrane oxygenation (ECMO).*

***Neonatal composite outcome:*** *stillbirth (≥20 weeks’ gestation); neonatal death (≤28 days post birth); neonatal seizures; necrotizing enterocolitis; hypoxic-ischemic encephalopathy; birthweight <3^rd^ centile (Hadlock); intubation or mechanical ventilation; non-invasive resuscitation; bacterial sepsis; intracranial hemorrhage; neonatal asphyxia; heart failure or cardiac arrest.*

**Table S2. Per-protocol sensitivity analysis, excluding women who changed antihypertensive agent**

|  | **Observed proportions** | | **Unadjusted effect estimates** | | **Adjusted effect estimates^a^** | |
| --- | --- | --- | --- | --- | --- | --- |
|  | **Labetalol (%)** | **Nifedipine (%)** | **Risk difference (95% CI) (%)** | **Risk ratio (95% CI)** | **Risk difference (95% CI) (%)** | **Risk ratio (95% CI)** |
| **Composite Maternal Outcome** | | | | | | |
| All hypertensive disorders | 395 (6.2) | 55 (9.8) | 3.61 (1.08, 6.14) | 1.58 (1.21, 2.07) | 3.00 (0.65, 5.35) | 1.46 (1.12, 1.91) |
| Preeclampsia^b^ | 238 (11.0) | 40 (17.3) | 6.28 (1.23, 11.3) | 1.57 (1.16, 2.13) | 6.29 (0.79, 11.8) | 1.57 (1.13, 2.19) |
| Gestational or chronic hypertension^b^ | 157 (3.7) | 15 (4.5) | 0.83 (-1.48, 3.14) | 1.22 (0.73, 2.05) | 0.65 (-1.81, 3.11) | 1.18 (0.66, 2.11) |
| **Composite Neonatal Outcome** | | | | | | |
| All hypertensive disorders | 2748 (42.3) | 253 (43.8) | 1.50 (-2.72, 5.72) | 1.04 (0.94, 1.14) | -1.43 (-5.71, 2.85) | 0.97 (0.87, 1.07) |
| Preeclampsia^b^ | 1139 (51.7) | 128 (52.2) | 0.59 (-6.00, 7.18) | 1.01 (0.89, 1.15) | -5.00 (-12.4, 2.38) | 0.90 (0.77, 1.06) |
| Gestational or chronic hypertension^b^ | 1609 (37.5) | 125 (37.5) | 0.08 (-5.32, 5.47) | 1.00 (0.87, 1.16) | -2.54 (-8.19, 3.12) | 0.93 (0.79, 1.10) |

*^a^ All models adjusted for the following: maternal age at delivery; maternal body mass index; marital status; conception via artificial reproductive technology; maternal diabetes mellitus (gestational or pre-existing); maternal pre-existing renal disease; smoking during pregnancy; socioeconomic status; plurality; and parity. Neonatal models additionally adjusted for gestational age at birth and mode of delivery. Per-protocol analysis excludes all crossover participants.*

***Maternal composite outcome:*** *maternal mortality (all-cause); eclampsia; HELLP syndrome; stroke; cortical blindness; retinal detachment; pulmonary oedema; placental abruption; liver rupture; renal failure requiring dialysis; heart or respiratory failure requiring extracorporeal membrane oxygenation (ECMO).*

***Neonatal composite outcome:*** *stillbirth (≥20 weeks’ gestation); neonatal death (≤28 days post birth); neonatal seizures; necrotizing enterocolitis; hypoxic-ischemic encephalopathy; birthweight <3^rd^ centile (Hadlock); intubation or mechanical ventilation; non-invasive resuscitation; bacterial sepsis; intracranial hemorrhage; neonatal asphyxia; heart failure or cardiac arrest.*

**Table S3. Sensitivity analysis, stratified by timing of randomisation**

|  | **Observed proportions** | | **Unadjusted effect estimates** | | **Adjusted effect estimates^a^** | |
| --- | --- | --- | --- | --- | --- | --- |
|  | **Labetalol (%)** | **Nifedipine (%)** | **Risk difference (95% CI) (%)** | **Risk ratio (95% CI)** | **Risk difference (95% CI) (%)** | **Risk ratio (95% CI)** |
| **Composite Maternal Outcome** | | | | | | |
| *11+0 – 19+6 weeks’ gestation* | 69 (4.9) | 13 (7.7) | 2.80 (-1.37, 6.97) | 1.57 (0.89, 2.78) | -0.17 (-2.15, 1.81) | 0.95 (0.65, 1.37) |
| *20+0 – 27+6 weeks’ gestation* | 87 (9.0) | 24 (16.6) | 7.59 (1.28, 13.90) | 1.85 (1.22, 2.80) | 4.67 (3.41, 5.94) | 1.57 (1.44, 1.71) |
| *28+0 – 36+6 weeks’ gestation* | 291 (6.7) | 26 (7.3) | 0.60 (-2.19, 3.38) | 1.09 (0.74, 1.60) | 1.26 (0.56, 1.95) | 1.19 (1.07, 1.32) |
| **Composite Neonatal Outcome** | | | | | | |
| *11+0 – 19+6 weeks’ gestation* | 558 (39.2) | 86 (50.0) | 10.84 (2.95, 18.73) | 1.28 (1.08, 1.50) | 2.28 (-6.77, 11.33) | 1.05 (0.85, 1.30) |
| *20+0 – 27+6 weeks’ gestation* | 457 (46.5) | 76 (49.7) | 3.18 (-5.33, 11.70) | 1.07 (0.90, 1.27) | 2.86 (-6.62, 12.34) | 1.06 (0.87, 1.28) |
| *28+0 – 36+6 weeks’ gestation* | 1933 (43.4) | 158 (43.1) | -0.39 (-5.66, 4.88) | 0.99 (0.88, 1.12) | -1.99 (-6.75, 2.76) | 0.95 (0.85, 1.07) |

*^a^ All models adjusted for the following: maternal age at delivery; maternal body mass index; marital status; conception via artificial reproductive technology; maternal diabetes mellitus (gestational or pre-existing); maternal pre-existing renal disease; smoking during pregnancy; socioeconomic status; plurality; and parity. Neonatal models additionally adjusted for gestational age at birth and mode of delivery.*

***Maternal composite outcome:*** *maternal mortality (all-cause); eclampsia; HELLP syndrome; stroke; cortical blindness; retinal detachment; pulmonary oedema; placental abruption; liver rupture; renal failure requiring dialysis; heart or respiratory failure requiring extracorporeal membrane oxygenation (ECMO).*

***Neonatal composite outcome:*** *stillbirth (≥20 weeks’ gestation); neonatal death (≤28 days post birth); neonatal seizures; necrotizing enterocolitis; hypoxic-ischemic encephalopathy; birthweight <3^rd^ centile (Hadlock); intubation or mechanical ventilation; non-invasive resuscitation; bacterial sepsis; intracranial hemorrhage; neonatal asphyxia; heart failure or cardiac arrest.*

**Table S4. Sensitivity analysis, stratified by timing of birth (neonatal outcomes)**

|  | **Observed proportions** | | **Unadjusted effect estimates** | | **Adjusted effect estimates^a^** | |
| --- | --- | --- | --- | --- | --- | --- |
|  | **Labetalol (%)** | **Nifedipine (%)** | **Risk difference (95% CI) (%)** | **Risk ratio (95% CI)** | **Risk difference (95% CI) (%)** | **Risk ratio (95% CI)** |
| **Composite Neonatal Outcome** | | | | | | |
| *<34 weeks’ gestation* | 516 (90.4) | 92 (93.9) | 3.51 (-1.82, 8.84) | 1.04 (0.98, 1.10) | 2.07 (-0.34, 4.49) | 1.02 (1.00, 1.05) |
| *≥34 weeks’ gestation* | 2432 (38.7) | 228 (38.4) | -0.30 (-4.39, 3.78) | 0.99 (0.89, 1.10) | -3.68 (-7.94, 0.57) | 0.90 (0.80, 1.02) |

*^a^ All models adjusted for the following: maternal age at delivery; maternal body mass index; marital status; conception via artificial reproductive technology; maternal diabetes mellitus (gestational or pre-existing); maternal pre-existing renal disease; smoking during pregnancy; socioeconomic status; plurality; and parity. Neonatal models additionally adjusted for gestational age at birth and mode of delivery.*

***Maternal composite outcome:*** *maternal mortality (all-cause); eclampsia; HELLP syndrome; stroke; cortical blindness; retinal detachment; pulmonary oedema; placental abruption; liver rupture; renal failure requiring dialysis; heart or respiratory failure requiring extracorporeal membrane oxygenation (ECMO).*

***Neonatal composite outcome:*** *stillbirth (≥20 weeks’ gestation); neonatal death (≤28 days post birth); neonatal seizures; necrotizing enterocolitis; hypoxic-ischemic encephalopathy; birthweight <3^rd^ centile (Hadlock); intubation or mechanical ventilation; non-invasive resuscitation; bacterial sepsis; intracranial hemorrhage; neonatal asphyxia; heart failure or cardiac arrest.*

**Table S5. Sensitivity analysis adjusting for year of delivery**

|  | **Observed proportions** | | **Unadjusted effect estimates** | | **Adjusted effect estimates^a^** | |
| --- | --- | --- | --- | --- | --- | --- |
|  | **Labetalol (%)** | **Nifedipine (%)** | **Risk difference (95% CI) (%)** | **Risk ratio (95% CI)** | **Risk difference (95% CI) (%)** | **Risk ratio (95% CI)** |
| **Composite Maternal Outcome** | | | | | | |
| All hypertensive disorders | 447 (6.6) | 63 (9.4) | 2.76 (0.48, 5.05) | 1.42 (1.10, 1.82) | 2.74 (0.23, 5.25) | 1.40 (1.05, 1.87) |
| Preeclampsia^b^ | 273 (11.8) | 43 (15.9) | 4.14 (-0.40, 8.71) | 1.35 (1.01, 1.82) | 4.50 (0.12, 8.88) | 1.37 (1.03, 1.81) |
| Gestational or chronic hypertension^b^ | 174 (3.9) | 20 (5.0) | 1.06 (-1.15, 3.26) | 1.27 (0.81, 1.99) | 1.57 (-0.83, 3.98) | 1.38 (0.83, 2.27) |
| **Composite Neonatal Outcome** | | | | | | |
| All hypertensive disorders | 2947 (43.0) | 318 (46.1) | 3.10 (-0.80, 7.00) | 1.07 (0.98, 1.17) | -2.41 (-6.37, 1.55) | 0.94 (0.86, 1.04) |
| Preeclampsia^b^ | 1261 (53.2) | 159 (55.6) | 2.39 (-3.71, 8.49) | 1.04 (0.94, 1.17) | -1.82 (-7.77, 4.12) | 0.96 (0.86, 1.08) |
| Gestational or chronic hypertension^b^ | 1686 (37.6) | 159 (39.4) | 1.76 (-3.21, 6.73) | 1.05 (0.92, 1.19) | -3.98 (-8.41. 0.45) | 0.89 (0.78, 1.02) |

*^a^All models adjusted for the following covariates: type of hypertension; maternal age at birth; maternal BMI; conception via artificial reproductive technology; maternal comorbidities (type 1 or 2 diabetes mellitus, renal disease, autoimmune disease); gestational diabetes mellitus; smoking during pregnancy; socioeconomic status; plurality; parity; and year of birth. Neonatal models also adjusted for mode of birth.*

*^b^Diagnosis at treatment initiation (randomization).*

***Maternal composite outcome:*** *Maternal mortality (all-cause); eclampsia; HELLP syndrome; stroke; cortical blindness; retinal detachment; pulmonary oedema; placental abruption; liver rupture; renal failure requiring dialysis; organ failure requiring extracorporeal membrane oxygenation.*

***Neonatal composite outcome:*** *Stillbirth (≥20 weeks’ gestation); neonatal death (≤28 days post-birth); neonatal seizures; necrotizing enterocolitis; hypoxic-ischemic encephalopathy; birthweight ≤3^rd^ centile (Hadlock); invasive resuscitation; non-invasive resuscitation; bacterial sepsis; intracranial hemorrhage; neonatal asphyxia; heart failure or cardiac arrest.*

**Table S6. Sensitivity analysis, excluding women with asthma**

|  | **Observed proportions** | | **Unadjusted effect estimates** | | **Adjusted effect estimates^a^** | |
| --- | --- | --- | --- | --- | --- | --- |
|  | **Labetalol (%)** | **Nifedipine (%)** | **Risk difference (95% CI) (%)** | **Risk ratio (95% CI)** | **Risk difference (95% CI) (%)** | **Risk ratio (95% CI)** |
| **Composite Maternal Outcome** | | | | | | |
| All hypertensive disorders | 394 (6.5) | 53 (9.7) | 3.21 (0.64, 5.78) | 1.49 (1.14, 1.96) | 2.67 (0.02, 5.32) | 1.41 (1.05, 1.90) |
| Preeclampsia^b^ | 241 (11.6) | 32 (15.4) | 3.83 (-1.27, 8.92) | 1.33 (0.95, 1.87) | 2.45 (-2.68, 7.58) | 1.22 (0.83, 1.77) |
| Gestational or chronic hypertension^b^ | 153 (3.9) | 21 (6.3) | 2.37 (-0.28, 5.03) | 1.61 (1.04, 2.51) | 2.83 (-0.16, 5.82) | 1.73 (1.09, 2.76) |
| **Composite Neonatal Outcome** | | | | | | |
| All hypertensive disorders | 2636 (43.0) | 251 (44.7) | 1.69 (-2.60, 5.98) | 1.04 (0.94, 1.14) | -1.10 (-5.76, 3.57) | 0.97 (0.87, 1.09) |
| Preeclampsia^b^ | 1131 (53.1) | 119 (53.6) | 0.55 (-6.34, 7.45) | 1.01 (0.89, 1.15) | -5.22 (-12.58, 1.82) | 0.90 (0.78, 1.04) |
| Gestational or chronic hypertension^b^ | 1505 (37.6) | 132 (38.8) | 1.22 (-4.18, 6.61) | 1.03 (0.90, 1.19) | 1.45 (-4.20, 7.10) | 1.04 (0.90, 1.20) |

*^a^All models adjusted for the following covariates: type of hypertension; maternal age at birth; maternal BMI; conception via artificial reproductive technology; maternal comorbidities (type 1 or 2 diabetes mellitus, renal disease, autoimmune disease); gestational diabetes mellitus; smoking during pregnancy; socioeconomic status; plurality; and parity. Neonatal models also adjusted for mode of birth.*

*^b^Diagnosis at treatment initiation (randomization).*

***Maternal composite outcome:*** *Maternal mortality (all-cause); eclampsia; HELLP syndrome; stroke; cortical blindness; retinal detachment; pulmonary oedema; placental abruption; liver rupture; renal failure requiring dialysis; organ failure requiring extracorporeal membrane oxygenation.*

***Neonatal composite outcome:*** *Stillbirth (≥20 weeks’ gestation); neonatal death (≤28 days post-birth); neonatal seizures; necrotizing enterocolitis; hypoxic-ischemic encephalopathy; birthweight ≤3^rd^ centile (Hadlock); invasive resuscitation; non-invasive resuscitation; bacterial sepsis; intracranial hemorrhage; neonatal asphyxia; heart failure or cardiac arrest.*

**Table S7. Sensitivity analysis, restricting to first pregnancy episode per woman**

|  | **Observed proportions** | | **Unadjusted effect estimates** | | **Adjusted effect estimates^a^** | |
| --- | --- | --- | --- | --- | --- | --- |
|  | **Labetalol (%)** | **Nifedipine (%)** | **Risk difference (95% CI) (%)** | **Risk ratio (95% CI)** | **Risk difference (95% CI) (%)** | **Risk ratio (95% CI)** |
| **Composite Maternal Outcome** | | | | | | |
| All hypertensive disorders | 414 (6.8) | 57 (9.5) | 2.69 (0.27, 5.11) | 1.40 (1.07, 1.82) | 2.17 (-0.21, 4.55) | 1.31 (0.98, 1.74) |
| Preeclampsia^b^ | 257 (11.9) | 41 (16.4) | 4.51 (-0.28, 9.30) | 1.40 (1.02, 1.87) | 3.41 (-0.96, 7.78) | 1.28 (0.94, 1.72) |
| Gestational or chronic hypertension^b^ | 157 (4.0) | 16 (4.5) | 0.57 (-1.68, 2.82) | 1.14 (0.69, 1.89) | 1.45 (-0.64, 3.53) | 1.34 (0.89, 2.03) |
| **Composite Neonatal Outcome** | | | | | | |
| All hypertensive disorders | 2646 (43.2) | 282 (46.8) | 3.55 (-0.62, 7.72) | 1.08 (0.99, 1.18) | -1.89 (-6.30, 2.52) | 0.96 (0.86, 1.06) |
| Preeclampsia^b^ | 1146 (53.0) | 138 (55.2) | 2.19 (-4.32, 8.71) | 1.04 (0.92, 1.17) | -2.28 (-8.76, 4.20) | 0.96 (0.84, 1.08) |
| Gestational or chronic hypertension^b^ | 1500 (37.9) | 144 (40.8) | 2.92 (-2.42, 8.27) | 1.08 (0.94, 1.23) | -2.77 (-7.92, 2.38) | 0.92 (0.80, 1.07) |

*^a^All models adjusted for the following covariates: type of hypertension; maternal age at birth; maternal BMI; conception via artificial reproductive technology; maternal comorbidities (type 1 or 2 diabetes mellitus, renal disease, autoimmune disease); gestational diabetes mellitus; smoking during pregnancy; socioeconomic status; plurality; and parity. Neonatal models also adjusted for mode of birth.*

*^b^Diagnosis at treatment initiation (randomization).*

***Maternal composite outcome:*** *Maternal mortality (all-cause); eclampsia; HELLP syndrome; stroke; cortical blindness; retinal detachment; pulmonary oedema; placental abruption; liver rupture; renal failure requiring dialysis; organ failure requiring extracorporeal membrane oxygenation.*

***Neonatal composite outcome:*** *Stillbirth (≥20 weeks’ gestation); neonatal death (≤28 days post-birth); neonatal seizures; necrotizing enterocolitis; hypoxic-ischemic encephalopathy; birthweight ≤3^rd^ centile (Hadlock); invasive resuscitation; non-invasive resuscitation; bacterial sepsis; intracranial hemorrhage; neonatal asphyxia; heart failure or cardiac arrest.*

**Table S8. Sensitivity analysis using propensity score matching**

|  | **Observed proportions** | | **Matched effect estimates** | |
| --- | --- | --- | --- | --- |
|  | **Labetalol (%)** | **Nifedipine (%)** | **Risk difference (95% CI) (%)** | **Risk ratio (95% CI)** |
| All hypertensive disorders | 35/614 (5.7) | 60/614 (9.8) | 4.07 (1.09, 7.05) | 1.71 (1.15, 2.56) |
| Preeclampsia | 25/241 (10.4) | 41/241 (17.0) | 6.64 (0.53, 12.75) | 1.64 (1.03, 2.61) |
| Gestational or chronic hypertension | 15/373 (4.0) | 19/373 (5.1) | 1.07 (-1.92, 4.06) | 1.27 (0.65, 2.45) |
| All hypertensive disorders | 276/631 (43.7) | 286/631 (45.3) | 1.58 (-3.91, 7.08) | 1.04 (0.92, 1.17) |
| Preeclampsia | 135/255 (52.9) | 136/255 (53.3) | 0.39 (-8.31, 9.09) | 1.01 (0.86, 1.19) |
| Gestational or chronic hypertension | 134/376 (35.6) | 150/37 (39.9) | 4.26 (-2.69, 11.20) | 1.12 (0.93, 1.35) |

*^a^Propensity score matching on the following covariates: type of hypertension; maternal age at birth; maternal BMI; conception via artificial reproductive technology; maternal comorbidities (type 1 or 2 diabetes mellitus, renal disease, autoimmune disease); smoking during pregnancy; socioeconomic status; plurality; and parity.*

*^b^Diagnosis at treatment initiation (randomization).*

*Propensity score matching in a 1:1 ratio with caliper set at 0.007.*

**Table S9. List of definitions used for primary and secondary outcomes**

| **Outcome** | **Definition (Including ICD-10 and ACHI codes)** |
| --- | --- |
| **Maternal Outcomes** | |
| Eclampsia | Convulsions in pregnant or puerperal women, associated with hypertension, oedema, and/or proteinuria. Captured in study datasets by ICD-10-AM code O15. |
| HELLP syndrome | A life-threatening complication of pregnancy characterized by hemolytic anemia, elevated liver enzymes, ad low platelet count. Captured in study datasets by ICD-10-AM code O14.2. |
| Stroke | A group of pathological conditions characterized by sudden, non-convulsive loss of neurological function due to brain ischemia or intracranial hemorrhage. Captured in study datasets by ICD-10-AM codes I60-I69 (cerebrovascular diseases). |
| Cortial blindness | Total loss of vision in all or part of the visual field due to bilateral occipital lobe damage or dysfunction. Captured in study datasets by ICD-10-AM code H47.61. |
| Retinal detachment | Detachment of retinal pigment epithelium. Captured in study datasets by ICD-10-AM code H33. |
| Pulmonary oedema | A buildup of fluid in the alveoli (air spaces) in the lungs. Captured in study datasets by ICD-10-AM code J81. |
| Placental abruption | Premature separation of the normally implanted placenta from the uterus. Captured in study datasets by ICD-10-AM code O45. |
| Liver rupture | A tear or break in the liver tissue. Captured in study datasets by ICD-10-AM code S36.11. |
| Renal failure requiring dialysis | An inability for kidneys to filter waste and excess fluid from the body effectively. Captured in study datasets by ICD-10-AM codes N17, O90.4, and Z92.2, and ACHI code 131000. |
| Extracorporeal membrane oxygenation (ECMO) | Life support therapy that takes over the function of the heart and/or lungs temporarily. Captured in study datasets by ACHI codes 9022500, 9022501, 9022502, and 3862702. |
| Requirement for additional antihypertensive(s) | Use of an antihypertensive (nifedipine, labetalol, or methyldopa) other than the allocated medication at any time following point of randomization. |
| Indicated birth | Non-spontaneous birth with an indication for delivery listed in the VPDC. |
| **Neonatal Outcomes** | |
| Stillbirth | Fetal death at or beyond 20 weeks’ gestation. Comprises both antepartum and intrapartum stillbirth. Captured in study datasets by ICD-10 codes O36.4 and P95, VPDC stillbirth flag, and NDI date of death. |
| Neonatal death | Death of a liveborn baby up to 28 days after birth. Captured in study datasets by ICD-10 code R95 and NDI date of death. |
| Neonatal seizures | Sudden, abnormal electrical discharges in the brain of an infant within the first 28 days of life. Captured in study datasets by ICD-10 codes P90 and G40. |
| Necrotizing enterocolitis | An inflammatory intestinal disease that causes sections of bowel tissue to become necrotic (die). Captured in study datasets by ICD-10 codes K55.3 and P77. |
| Hypoxic-ischemic encephalopathy | A condition which occurs when a newborn’s brain is deprived of blood and/or oxygen, causing damage and swelling. Captured in study datasets by ICD-10 code P91.6. |
| Birthweight ≤ 3^rd^ centile | Calculated using Hadlock birthweight centile. |
| Invasive resuscitation (intubation or mechanical ventilation) | Use of invasive resuscitation such as endotracheal intubation or assisted ventilation. Captured in study datasets by ICD-10 codes Z99.1 and Z99.8, ACHI codes 2200701, 2200800-01, 1388201-02, 9203500-01, 9204100, 921100, 9017902, and 9017905, and VPDC indicator for invasive resuscitation. |
| Non-invasive resuscitation | Other methods of resuscitation including oxygen therapy, positive airway pressure, cardiopulmonary resuscitation, or medications (e.g., narcotic antagonists, adrenaline, sodium bicarbonate). Captured in study datasets by ACHI codes 9024200 and 9205200 and VPDC indicator for non-invasive resuscitation. |
| Neonatal sepsis | A blood infection in infants under 28 days old. Captured in study datasets by ICD-10 code P36. |
| Intracranial hemorrhage | A birth injury that causes blood to accumulate within the cranial vault. Captured in study datasets by ICD-10 codes P52 and P10. |
| Neonatal asphyxia | A condition where a newborn is deprived of oxygen and blood flow. Captured in study datasets by ICD-10 codes P24, T79, and R09.0. |
| Heart failure or cardiac arrest | A condition where a) a newborn’s heart is unable to keep up with the demands of the body (heart failure) or b) there is a sudden cessation of heart function (cardiac arrest). Captured in study datasets by ICD-10 codes I46, P29.0, and P29.81. |
| Iatrogenic preterm birth | Any non-spontaneous birth occurring prior to 37 weeks’ gestation. Captured in study datasets by calculating gestational age at birth. |
| Major congenital anomaly | Please refer to EURO-CAT guidelines for a list of relevant ICD-10 codes. |
| Neonatal hypoglycaemia | A condition where a newborn’s blood sugar (glucose) is lower than normal. Captured in study datasets by ICD-10 code P70. |
| Neonatal hypothermia | A condition wherein a newborn’s body temperature drops below 36.5ºC. Captured in study datasets by ICD-10 codes P80, T68, and 6A4. |
| Respiratory distress | A breathing problem that occurs in infants due to a lack of surfactant in the lungs. Captured in study datasets by ICD-10 codes P22, J80, and P28.5. |

**Table S10. E-value for point estimates and 95% confidence interval closest to the null value for the risk ratios (RR) and risk differences (RD) of statistically significant primary and secondary outcomes**

| **Outcome** | **RR E-value (95% CI E-value)** | **RD E-value (95% CI E-value)** |
| --- | --- | --- |
| **Primary Outcomes** | | |
| Maternal primary outcome (all hypertensive disorders) | 1.99 (1.34) | 2.40 (1.54) |
| Maternal primary outcome (preeclampsia) | 1.97 (1.71) | 2.04 (1.00) |
| **Secondary Outcomes** | | |
| Requirement for additional antihypertensives | 4.13 (3.83) | 3.42 (3.00) |
| Iatrogenic preterm birth <37 weeks’ gestation | 1.83 (1.57) | 1.77 (1.42) |
| Iatrogenic preterm birth <34 weeks’ gestation | 2.41 (2.24) | 2.48 (1.77) |
| Iatrogenic preterm birth <32 weeks’ gestation | 2.62 (2.06) | 2.91 (1.83) |
| Iatrogenic preterm birth <28 weeks’ gestation | 4.21 (3.50) | 3.70 (1.60) |
| Neonatal hypoglycaemia | 1.85 (1.70) | 1.32 (1.00) |
